# The CannTeen study: verbal episodic memory, spatial working memory, and response inhibition in adolescent and adult cannabis users and age-matched controls

**DOI:** 10.1007/s00213-022-06143-3

**Published:** 2022-04-29

**Authors:** W. Lawn, N. Fernandez-Vinson, C. Mokrysz, G. Hogg, R. Lees, K. Trinci, K. Petrilli, A. Borissova, S. Ofori, S. Waters, P. Michór, M. B. Wall, T. P. Freeman, H. V. Curran

**Affiliations:** 1 Clinical Psychopharmacology Unit, University College London, London, UK; 2 Department of Addictions, Institute of Psychiatry, Psychology and Neuroscience, King’s College London, London, UK; 3 Department of Psychology, Institute of Psychiatry, Psychology and Neuroscience, King’s College London, London, UK; 4 Faculty of Biology, Medicine and Health, University of Manchester, Manchester, UK; 5 Addiction and Mental Health Group (AIM), Department of Psychology, University of Bath, Bath, UK; 6 Department of Neuroimaging, Institute of Psychiatry, Psychology and Neuroscience, King’s College London, London, UK; 7 NIHR University College London Hospitals Biomedical Research Centre, University College Hospital, London, UK; 8 Department of Psychiatry, University of Oxford, Warneford Hospital, Oxford, UK; 9 School of Life Sciences, University of Warwick, Coventry, UK; 10 Invicro London, Hammersmith Hospital, Burlington Danes Building, Du Cane Road, London, UK

**Keywords:** Cannabis, Marijuana, Adolescence, Response inhibition, Memory, Working memory, Verbal memory, Episodic memory, Cognition

## Abstract

**Background:**

Preclinical and human studies suggest that adolescent cannabis use may be associated with worse cognitive outcomes than adult cannabis use. We investigated the associations between chronic cannabis use and cognitive function in adolescent and adult cannabis users and controls. We hypothesised user-status would be negatively associated with cognitive function and this relationship would be stronger in adolescents than adults.

**Methods:**

As part of the ‘CannTeen’ project, this cross-sectional study assessed cognitive performance in adolescent cannabis users (*n* = 76; 16–17-year-olds), adolescent controls (*n* = 63), adult cannabis users (*n* = 71; 26–29-year-olds) and adult controls (*n* = 64). Users used cannabis 1–7 days/week. Adolescent and adult cannabis users were matched on cannabis use frequency (4 days/week) and time since last use (2.5 days). Verbal episodic memory (VEM) was assessed using the prose recall task, spatial working memory (SWM) was assessed using the spatial n-back task, and response inhibition was assessed with the stop-signal task. Primary outcome variables were: delayed recall, 3-back discriminability, and stop signal reaction time, respectively.

**Results:**

Users had worse VEM than controls (*F*(1,268) = 7.423, *p* = 0.007). There were no significant differences between user-groups on SWM or response inhibition. Null differences were supported by Bayesian analyses. No significant interactions between age-group and user-group were found for VEM, SWM, or response inhibition.

**Conclusions:**

Consistent with previous research, there was an association between chronic cannabis use and poorer VEM, but chronic cannabis use was not associated with SWM or response inhibition. We did not find evidence for heightened adolescent vulnerability to cannabis-related cognitive impairment.

**Supplementary Information:**

The online version contains supplementary material available at 10.1007/s00213-022-06143-3.

## Introduction

Cannabis is the most commonly used internationally controlled drug by adolescents, with 19% of English 15 year-olds (NHS-Digital 2018) and 28% of American 15–16 year-olds (NIDA 2020) reporting past-year use. Long-term cannabis use has been linked to compromised function in some cognitive domains (Broyd et al. 2016; Curran et al. 2016; Lovell et al. 2020; Figueiredo et al. (2020). Predicated on continuing brain development (Blakemore and Choudhury 2006; Dumontheil 2016), the cognitive consequences of adolescent cannabis use are thought to be more severe and enduring than those caused by adult use (Blest-Hopley et al. 2020; Meier et al. 2012). However, direct comparisons between adolescents and adults are lacking.

Throughout adolescence, cognitive abilities are refined as the brain undergoes neurobiological changes, including synaptic pruning and maturation of the endocannabinoid (eCB) system (Ellgren et al. 2008; Larsen and Luna 2018; Rubino and Parolaro 2016; Verdurand et al. 2011). The eCB system is involved in neuronal reorganisation and the development of cognitive, reward, and executive control brain systems (Fischer et al. 2020; Galve-Roperh et al. 2009; Lu and MacKie 2016). Chronic adolescent cannabis exposure may disrupt this maturation (Ellgren et al. 2008; Rubino et al. 2015; Verdurand et al. 2011), which may consequently impair cognition.

Non-human studies of learning and memory have shown adolescent exposure to delta-9-tetrahydrocannabinol (THC) and cannabinoid agonists lead to adult impairments (Rubino & Parolaro 2016; Verrico et al. 2014). Some research suggests that adolescent rodents experience greater harm than adult rodents (O’shea et al. 2004; Quinn et al. 2008; Schneider and Koch 2003), although recovery after adolescent THC administration to primates has been reported (Verrico et al. 2020). If, and in which domains, human adolescents suffer worse cognitive impacts than adults remains unknown. Our choices of the cognitive domains assessed, introduced below, were informed by the extant literature and our previous acute cannabis administration research in adolescents (Mokrysz et al. 2016).

Episodic memory is defined as declarative memory for past events and experiences (Tulving 2002). Verbal episodic memory (VEM) is memory for episodic information that has been presented verbally. Cross-sectional studies have found significantly worse verbal recall in both adolescent (Harvey et al. 2007; Solowij et al. 2011) and adult (Gonzalez et al. 2012) cannabis users, compared to non-users. Additionally, an earlier age of cannabis use onset has been associated with greater verbal recall impairment (Becker et al. 2018; Solowij et al. 2011) and escalating cannabis use in adolescence predicted worse immediate verbal recall (Duperrouzel et al. 2019). Indeed, a meta-analysis revealed that cannabis users performed significantly worse than non-users in both immediate and delayed verbal recall (Schoeler et al. 2016). In sum, after abstinence periods of at least 12–24 h, both adult and adolescent cannabis users appear to have impaired VEM relative to controls. However, whether adolescents are at greater risk of experiencing these VEM deficits is unknown.

Working memory refers to the temporary storage and manipulation of information necessary to keep things in mind while performing complex tasks (Baddeley 2010; Chai et al. 2018). Within the multicomponent model of working memory, spatial working memory (SWM) can be defined as the ability to link a visual stimulus to a specific location (Cowan et al. 2006), and is often measured using the spatial n-back task (Green et al. 2005). Much of the previous research in adults has reported null relationships between chronic cannabis use and SWM ability, following > 24 h abstinence (Cousijn et al. 2014a; 2014b; Desrosiers et al. 2015) and after no abstinence (Gonzalez et al. 2012). However, in a study of nearly 4,000 young adolescents, there was some evidence that cannabis use was associated with compromised SWM capacity (Morin et al. 2019), and more so in females than males (Noorbakhsh et al. 2020). Longitudinal twin studies of young people have revealed contradictory findings regarding the impact of cannabis use over-and-above genetic and familial factors on working memory and other executive functions (Meier et al. 2018; Ross et al. 2020). Other studies in adolescents have been mixed, with some reporting negative associations between cannabis use and SWM (Becker et al. 2018; Harvey et al. 2007; Tervo-Clemmens et al. 2018). A meta-analysis of cognitive function across different domains in adolescent and young adults found that working memory was reduced in users compared to controls (Scott et al. 2018), but this was not found in a meta-analysis of regular adult users (Lovell et al. 2020). Hence, the extant literature tentatively implies that adolescent, but not adult, users may show cannabis-related reductions in SWM capacity, but this is as yet untested.

Response inhibition is the ability to inhibit an action that is no longer appropriate or desired in a specific context, which is crucial for goal-directed behaviour and executive control (Verbruggen and Logan 2008). Cross-sectional studies have found no significant differences between adult users and non-users in stop signal reaction time (SSRT) (Gonzalez et al. 2012; Grant et al. 2012) and go/no-go task performance (Hester et al. 2009; Wallace et al. 2020) after varying abstinence periods. Indeed, a meta-analysis reported chronic cannabis use was not associated with motor impulsivity (Figueiredo et al. 2020). However, one study reported worse response inhibition performance in cannabis users relative to controls (Moreno et al. 2012) and a meta-analysis in young people found an association between cannabis use and inhibition, in general (Scott et al. 2018). Hence, more behavioural research into response inhibition in cannabis-using adolescents is needed.

Predicated on continued neuropsychological development and some, but not consistent, age-of-onset effects, adolescents were hypothesised to be more vulnerable to the harmful effects of long-term cannabis on cognitive function. When previous research has investigated moderation by age, it has examined the impact of adolescent use on subsequent adult cognition. However, to our knowledge, no studies have directly compared adolescent and adult current cannabis users and controls, while ensuring they are matched on age, gender, and cannabis use frequency. Therefore, the differential, contemporary impact on cognitive function of non-acute cannabis use in adolescents and adults is undetermined.

### Aims and hypotheses

In this study, we investigated the relationship between *current* cannabis use and VEM, SWM, and response inhibition in matched adolescents and adults. As registered on the Open Science Framework (OSF; (Lawn et al. 2021), our hypotheses were:

1. Cannabis users will have poorer VEM, SWM and response inhibition than controls.
2. There will be user-group by age-group interactions on VEM, SWM and response inhibition, where the user vs. control difference will be greater in adolescents than in adults.
3. Within users, there will be negative associations between frequency of use and task performance, and the relationships will be stronger in adolescents than adult users.

For each hypothesis, we also predicted that associations will persist after adjusting for pre-defined covariates (Lawn et al. 2021).

## Methods

### Design and participants

This is a cross-sectional analysis of baseline data from the longitudinal ‘CannTeen’ study. The study protocol (Lawn et al. 2020) describes the methods of the project in full. Ethical approval was obtained from the University College London (UCL) ethics committee (project ID 5929/003). All participants provided written, informed consent, and this study was conducted in line with the Declaration of Helsinki.

The full sample comprised 274 participants: 76 adolescent users, 71 adult users, 63 adolescent controls, and 64 adult controls. Adolescent users and controls, and adult users and controls, were both matched on age and gender; and the two user-groups were matched on cannabis-use frequency.

For full eligibility criteria, see the supplementary materials. In brief, for adolescent users, inclusion criteria were: aged 16–17 years; use cannabis 1–7 days/week. For adult users, inclusion criteria were: aged 26–29 years; use cannabis 1–7 days/week; and exclusion criteria were: having used cannabis on a weekly or more frequent basis before age 18 years. For adolescent controls, inclusion criteria were: aged 16–17 years; have used cannabis or tobacco at least once but no more than 10 lifetime uses of cannabis. For adult controls, inclusion criteria were aged 26–29 years; have used cannabis or tobacco at least once but no more than 10 lifetime uses of cannabis.

Exclusion criteria for all participants were current treatment for a mental health disorder; current daily use of any psychotropic medication; a personal history of psychotic disorder; or use of any illicit drug except cannabis more than twice per month.

We recruited controls with limited cannabis or tobacco exposure, rather than people with no exposure, with the aim of more closely matching the controls and users on the opportunity to use drugs and associated unmeasured confounding variables.

Participants were recruited from online adverts, school assemblies, university campus posters, public posters and flyers, and word-of-mouth.

### Measures

#### Prose recall task

VEM was assessed using the prose recall task from the Rivermead Behavioural Memory Test battery (Wilson et al. 1989). Participants were played a 30-s story via headphones, after which they immediately wrote down what they could remember (i.e. immediate recall). After an approximately 20-min delay filled with unrelated assessments, participants again wrote down what they could remember from the story (i.e. delayed recall). The story contained 21 ‘idea units’. For each idea unit, one point was given for a word-perfect recall or exact synonym, and half a point was given for a partial recall or close synonym. The maximum score was therefore 21. The primary outcome variable was delayed recall, and the secondary outcome variable was immediate recall.

#### Spatial N-back task

The spatial n-back task was used as an assessment of SWM (Green et al. 2005). This task was run with PsychoPy software (Peirce et al. 2019). In brief, participants responded to a blue square which appeared sequentially in one of six locations on the screen. They responded ‘yes’ or ‘no’ as to whether the square was: (1) in the 12 o’clock’ position (0-back condition), (2) in the same position as the square in the previous trial (1-back condition), (3) in the same position as the square two trials before (2-back condition), (4) in the same position as the square three trials before (3-back condition). We calculated performance at each load: 0-back, 1-back, 2-back and 3-back. The most sensitive and specific outcome measure of n-back performance is discriminability (d') (Haatveit et al. 2010) (d′ = Z_Proportion of Hits_–Z_Proportions of False Alarm_). d’ on the 3-back condition was the task’s primary outcome variable. See supplementary materials for a full task description, secondary outcome variables, and reasons for exclusion of datapoints.

#### Stop signal task

To measure response inhibition, the stop signal task (Verbruggen et al. 2008) was employed. The task was run with PsychoPy software (Peirce et al. 2019). In brief, a series of white arrows appeared sequentially on the screen and participants responded by pressing the appropriate left or right arrow key (go trials). However, on 25% of the trials, after a variable delay, the arrow turned blue, and participants tried to inhibit their response (stop trials). Staircase tracking of the stop signal delay (SSD) time occurred so that each participant had a ~ 50% chance of successful response inhibition, ensuring a reliable stop signal reaction time (SSRT) was calculated, which was the task’s primary outcome variable (Verbruggen et al. 2019; Verbruggen & Logan 2008) (SSRT = mean reaction time on go trials – mean SSD). See supplementary materials for a full task description, secondary outcome variables, and reasons for exclusion of datapoints.

#### Cannabis use measures

We used a timeline follow-back (TLFB) method (Robinson et al. 2014) to record drug use over the past 12 weeks. We used the TLFB data to quantify cannabis use frequency (in days/week), days since last use of cannabis, most common type of cannabis used (‘strong’ herbal; ‘weak’ herbal; and ‘hash’, see supplementary materials) (Freeman & Winstock 2015). The TLFB method has been approved by expert consensus for measuring cannabis use (Lorenzetti et al. 2021). Users also reported the age at which they first used cannabis, when they first started using weekly, and how many grams they used on a day of use. The duration of weekly cannabis use was calculated by subtracting the age at which they started using weekly from their current age. Controls reported if they had ever used cannabis and, if so, how many times they had used cannabis in their life. We also collected cannabis samples from a small subsample (*n* = 26) of users to quantify average THC concentration in cannabis used by adolescents and adults.

#### Other measures and pre-defined covariates

Other measures included the alcohol use disorder identification test (AUDIT) (Babor et al. 2001), cannabis use disorder identification test-revised (CUDIT-R) (Adamson et al. 2010), breathalyser and saliva drugs tests. See the supplementary materials for detailed information on these measures.

We included pre-defined covariates in analyses in order to adjust for variables which are thought to be theoretically related to outcomes, and to possibly differ by group. Pre-defined covariates were gender, socio-economic status, risk-taking level (De Haan et al. 2011), premorbid verbal intelligence (Holdnack 2001), daily tobacco use, twice-weekly alcohol use, and monthly illicit drug (see supplementary materials). The final three covariates were all measured using the TLFB (Robinson et al. 2014).

### Procedure

As described in the full protocol (Lawn et al. 2020), interested participants were initially pre-screened using an online questionnaire and subsequently screened on the telephone to assess eligibility. Potentially eligible participants were invited to UCL to complete their baseline session. Further eligibility criteria were assessed at the start of the baseline session, including body mass index, an official identification check to verify age, and saliva drugs tests. Breathalyzer tests and self-report were used to confirm recent cannabis (> 12 h), alcohol (> 12 h), and other illicit drug (> 48 h) abstinence. The majority of the cannabis users were not daily users; the sample drank infrequently; and they used illicit drugs infrequently, therefore these abstinence requirements did not necessitate substantial behavioural change in order to participate. This explains why the average time since last use of cannabis (Table 2) is considerably longer than the required minimum abstinence period of 12 h. The baseline session then continued with various cognitive, mental health, and behavioural measures, including those described above; the others will be reported elsewhere.

### Statistical power

The project was not powered specifically for this analysis. The project was powered to detect a cross-sectional group difference in cannabis use disorder between adolescent and adult cannabis users, with an odds ratio effect size of three. However, a power calculation based on our number of participants (*n* = 274) indicated that we had 80% power to detect small age-group by user-group interactions, of size Cohen’s f ≥ 0.17, at an alpha value of 0.05.

### Statistical Analyses

Statistical tests were conducted on IBM SPSS Statistics Version 27. Assumptions for parametric analyses were checked (see supplementary materials).

For analyses of primary outcomes (delayed recall, d’ on 3-back, SSRT), we ran 2 × 2 between-subjects factorial analyses of variance (ANOVAs). Between-subjects factors were age-group and user-group. Significant interactions were followed up with Bonferroni-corrected post-hoc pairwise *t*-tests. For primary outcome variables, analyses of covariance (ANCOVAs) were then run with the pre-defined covariates included. Subsequently, ANCOVAs were also run in the user-group only, with a between-subjects factor of age-group and a covariate of cannabis use frequency (days/week) to investigate relationships between the primary outcome variables and cannabis use frequency. We then included pre-defined covariates in these ANCOVAs. For primary outcome variables, when results were non-significant, post-hoc Bayesian independent-samples tests were run to compare users with controls, and to compare adolescent users with adolescent controls. We assumed equal variances and used a Jeffreys default prior. Bayes factors (BF_01_) ≥ 3 support the null hypothesis of no difference.

For prose recall, we also performed an exploratory 2 × 2 × 2 mixed ANOVA with between-subjects factors of age-group and user-group and a within-subjects factor of time (immediate, delayed). As an additional exploratory analysis, only in adult users, we conducted Pearson correlations between age-of-onset and each primary outcome variable.

For secondary outcome variables (see supplementary materials), we conducted 2 × 2 ANOVAs with between-subjects factors of age-group and user-group. When data did not meet assumptions for parametric analyses, we supplemented these with non-parametric Mann–Whitney *U*-tests.

## Results

### Participant characteristics (Tables 1 and 2)

Demographic and cannabis use variables from the full sample of 274 participants are presented in Tables 1 and 2. In brief, groups were matched on gender and ethnicity. Adolescent users (17.1 years) and controls (17.1 years) and adult users (27.6 years) and controls (27.4 years) were matched on age. Adolescent users (3.7 days/week; 2.4 days since use) and adult users (4.1 days/week; 2.5 days since use) were matched on cannabis use frequency and days since last use. A similar number of adolescent users (*n* = 69, 90.8%) and adult users (*n* = 59, 83.1%) used strong herbal (i.e. ‘skunk’) cannabis as their most common type of cannabis (Table 2). Furthermore, albeit in a small subsample, the adolescent users (21.1%, SD = 5.2, *n* = 14) and adult users (21.3%, SD = 4.6, *n* = 12), used strong herbal cannabis of a similar THC concentration. For full details see supplementary materials.

**Table 1:** Sociodemographic characteristics of full sample (*n* = 274). AUDIT is the alcohol use disorders identification test. RT-18 is Risk-Taking-18. SES is socioeconomic status. WTAR is Wechsler test of adult reading. Daily tobacco refers to non-cannabis cigarettes and roll-ups. Ethnicity is compared using white vs. non-white. WTAR data were missing for 2 adolescent users and 2 adult users. One ethnicity datapoint was missing for adolescent users. SES data were missing for one adolescent user, one adolescent control, three adult users, and one adult control. Continuous data are presented as mean [SD], and categorical data are presented as *n* (%). Group differences are highlighted in the final column, *p < 0.05, **p < 0.01. ***p < 0.001

	Adolescent user (*n* = 76)	Adolescent control (*n* = 63)	Adult user (*n* = 71)	Adult control (*n* = 64)	Group differences
*Gender:*					
Male	38 (50.0%)	31 (49.2%)	38 (53.5%)	31 (48.4%)	
Female	38 (50.0%)	32 (50.8%)	33 (46.5%)	33 (51.6%)	
*Age (years)*	17.1 [0.5]	17.1 [0.5]	27.6 [1.2]	27.4 [1.0]	Adult > adolescent***
*Ethnicity:*					
White	51 (68.0%)	40 (63.5%)	45 (63.4%)	41 (64.1%)	
Mixed	15 (20.0%)	7 (11.1%)	8 (11.3%)	3 (4.7%)	
Asian	2 (2.7%)	10 (15.9)%	11 (15.5%)	15 (23.4%)	
Black	4 (5.3%)	2 (3.2%)	6 (8.5%)	2 (3.1%)	
Other	3 (4.0%)	2 (3.2%)	1 (1.4%)	2 (3.1%)	
Prefer not to say	0 (0.0%)	2 (3.2%)	0 (0.0%)	1 (1.6%)	
Alcohol use frequency (days/week)	0.6 [0.6]	0.7 [0.8]	1.5 [1.4]	1.4 [1.0]	Adult > adolescent***
AUDIT	6.5 [4.6]	4.3 [3.5]	6.0 [4.3]	5.5 [4.2]	User > control**
Use Tobacco Daily (yes)	10 (13.2%)	2 (3.2%)	9 (12.7%)	2 (3.1%)	Users > control**
Use another illicit drug at least once a month (yes)	45 (59.2%)	2 (3.2%)	18 (25.4%)	1 (1.6%)	Users > controls*** Adolescent users > adult users***
*SES:*					
Mother’s education undergraduate degree or above	44 (58.7%)	36 (58.1%)	31 (45.6%)	27 (42.9%)	Adolescent > adult*
*RT-18*	11.4 (3.1)	9.1 (4.1)	8.8 (3.9)	7.6 (4.1)	User > control*** Adolescent > adult***
*WTAR adjusted*	111.6 [9.2]	110.5 [10.5]	107.0 [9.9]	110.5 [9.6]

**Table 2:** Cannabis use variables for adolescent users and adult users. Continuous data are presented as mean [SD], and categorical data are presented as *n* (%). Data for three adult users are missing for amount of cannabis used on a day of use (for users). Group differences are highlighted in the final column *p < 0.05, **p < 0.01, ***p < 0.001

	Adolescent User (*n* = 76)	Adult User (*n* = 71)	Adolescent Control (*n* = 64)	Adult Control (*n* = 64)	Group Differences
Days since last cannabis use	2.4 [2.6]	2.5 [4.6]	n/a	n/a	
Age (years) of first cannabis use	14.6 [1.1]	18.0 [2.9]	n/a	n/a	Adult user > adolescent user*
Cannabis use frequency (days/week)	3.7 [2.0]	4.1 [1.9]	n/a	n/a	
Number of users who most commonly use strong herbal cannabis (i.e. ‘skunk’)	69 (90.8%)	59 (83.1%)	n/a	n/a	
Grams of cannabis used on a day when using	1.1 [0.8]	0.6 [0.7]	n/a	n/a	Adolescent user > adult user*
Duration of weekly cannabis use (years)	1.5 [0.9]	5.3 [2.7]	n/a	n/a	Adult user > adolescent user***
CUDIT	15.4 [5.6]	11.9 [4.8]	n/a	n/a	Adolescent user > adult user***
Ever used cannabis (yes)	n/a	n/a	55 (87.3%)	62 (96.9%)	Adult control > adolescent control*
Number of lifetime cannabis uses	n/a	n/a	3.4 [2.8]	4.5 [3.1]	Adult control > adolescent control*

### Prose recall task—delayed recall (Fig. 1 & Table 3)

**Fig. 1:**
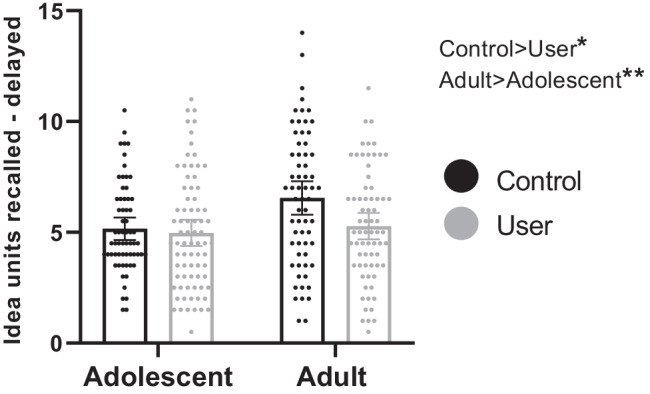
Mean idea units recalled at the delayed time point with datapoints overlaid, for adolescent users (*n* = 76), adolescent controls (*n* = 63), adult users (*n* = 69), and adult controls (*n* = 64). Error bars represent 95% confidence intervals. Main effect of age-group was significant at *p* < 0.01**; main effect of user-group was significant at *p* < 0.05*; the interaction was trend at *p* = 0.084, but not robust to inclusion of covariates

**Table 3:** Mean values for the prose recall (delayed recall), stop signal task (stop signal reaction time - SSRT) and spatial n-back (3-back discriminability) primary outcome variables across the four groups. Numbers of participants in each group vary due to exclusion of participants where performance did not meet criteria. 95% confidence intervals (CI) are shown in brackets

	Adolescent user	Adolescent control	Adult user	Adult control
Prose recall	*n* = 76	*n* = 63	*n* = 69	*n* = 64
Delayed Recall	4.974 [4.383—5.565]	5.167 [4.660—5.673]	5.283 [4.688—5.886]	6.555 [5.800—7.310]
Spatial n-back	*n* = 62	*n* = 59	*n* = 58	*n* = 58
Mean 3-back *d'*	1.325 [1.113—1.537]	1.262 [1.057—1.467]	1.291 [1.081—1.501]	1.413 [1.168—1.659]
Stop Signal	*n* = 72	*n* = 55	*n* = 67	*n* = 62
Mean SSRT	0.264 [0.253—0.275]	0.257 [0.247—0.267]	0.251 [0.243—0.260]	0.252 [0.240—0.263]

Two adult users were excluded from prose recall analyses because they received the wrong prose recall story, sample sizes were as follows: adolescent users *n* = 76; adolescent controls *n* = 63; adult users *n* = 69; adult controls *n* = 64 (Table 3). Mean values for delayed recall are presented in Table 3.

The interaction between age-group and user-group was non-significant, but at a trend level (*F*(1,268) = 3.002, *p* = 0.084, η_p_^2^ = 0.011). Exploration of this trend interaction showed that within adults, users performed worse than controls (*t*(131) = 2.865, *p* = 0.005, MD = 1.272), but within adolescents the difference was non-significant (*t*(137) = 0.443, *p* = 0.659, MD = 0.193).

There was a significant main effect of age-group (*F*(1,268) = 7.423, *p* = 0.007, η_p_^2^ = 0.027), with adults (mean = 5.89, SD = 2.83) recalling more than adolescents (mean = 5.06, SD = 2.34). There was also a significant main effect of user-group (*F*(1,268) = 5.533, *p* = 0.019, η_p_^2^ = 0.020), with controls (mean = 5.87, SD = 2.65) recalling more than users (mean = 5.12, SD = 2.55). With inclusion of covariates, the significant main effect of age-group persisted, and the trend interaction became less significant (*F*(1,252) = 1.547, *p* = 0.215, η_p_^2^ = 0.006; see supplementary table S2). The main effect of user-group became narrowly non-significant (*F*(1,252) = 3.780, *p* = 0.053, η_p_^2^ = 0.015).

In users only, the interaction between number of cannabis use days/week and age-group was non-significant (*F*(1,141) = 0.025, *p* = 0.874, η_p_^2^ < 0.001). There was a non-significant, trend main effect of cannabis use frequency on delayed prose recall (*F*(1,141) = 3.276, *p* = 0.072, η_p_^2^ = 0.023). Inclusion of covariates did not change this pattern of results (see supplementary table S3).

For immediate recall results, see supplementary tables S4 and S5. In brief, the pattern of results for immediate recall was the same as for delayed recall. For the 2 × 2 × 2 mixed ANOVA in which time was included as a within-subjects factor, see supplementary table S11. In brief, immediate recall was better than delayed recall. However, time did not interact with age-group or user-group significantly, and the pattern of results remained similar.

### Spatial n-back task—3-back d’ (Fig. 2 and Table 3)

**Fig. 2:**
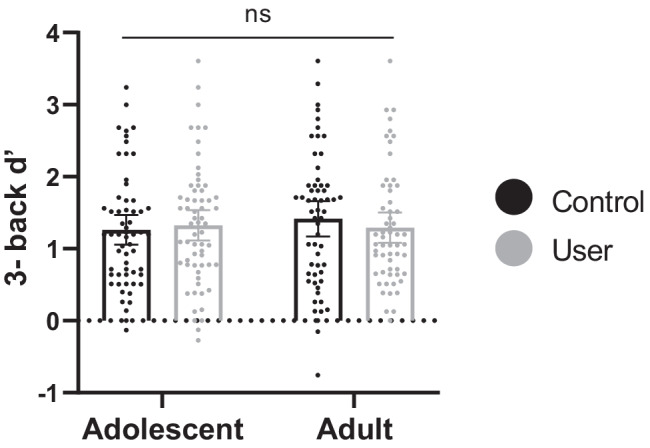
Mean 3-back *d’* with datapoints overlaid, for adolescent users (*n* = 62), adolescent controls (*n* = 59), adult users (*n* = 58), and adult controls (*n* = 58). Error bars represent 95% confidence intervals. Both age-group and user-group main effects were non-significant, and the interaction was non-significant (ns)

In line with the spatial n-back exclusion criteria, 30 participants were excluded (see supplementary materials). A further 7 participants were excluded due to missing data. After these participants were excluded, sample sizes were as follows: adolescent users *n* = 62; adolescent controls *n* = 59; adult users *n* = 58; adult controls *n* = 58 (Table 3).

The interaction between age-group and user-group was non-significant (*F*(1,233) = 0.722, *p* = 0.396, η_p_^2^ = 0.003). Main effects of age-group (*F*(1,233) = 0.289, *p* = 0.591, η_p_^2^ = 0.001) and user-group (*F*(1,233) = 0.075, *p* = 0.785, η_p_^2^ < 0.001) were non-significant. This pattern of results was not changed by inclusion of covariates in the ANCOVA (see supplementary table S2). Post hoc Bayesian analyses supported the null hypothesis of no differences in 3-back d’ between user-groups (BF_01_ = 9.480) and between adolescent users and adolescent controls (BF_01_ = 6.519). See Fig. 1 for mean 3-back d’ in each group. For secondary outcome variable results, see supplementary tables S6 and S7. In brief, all but two tests were non-significant, demonstrating no meaningful differences between the groups on the spatial n-back task.

In users only, the interaction between age-group and cannabis use frequency was non-significant (*F*(1,116) = 0.313, *p* = 0.577, η_p_^2^ = 0.003). The main effect of cannabis use frequency (*F*(1,116) = 1.138, *p* = 0.288, η_p_^2^ = 0.010) was also non-significant. This pattern of results was unchanged by inclusion of covariates in the ANOVA (see supplementary table S3).

### Stop signal task—SSRT (Fig. 3 and Table 3)

**Fig. 3:**
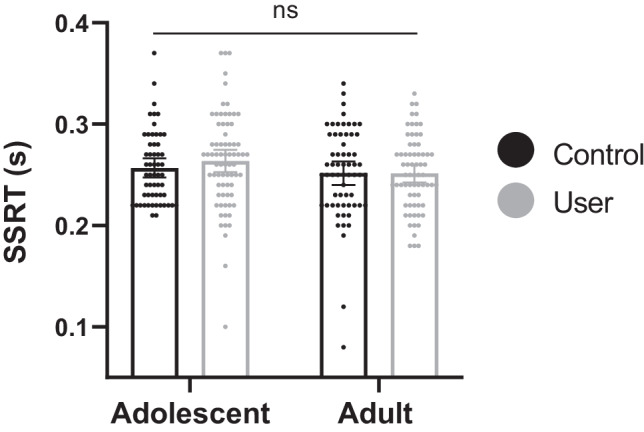
Mean SSRT (in seconds) with datapoints overlaid, for adolescent users (*n* = 72), adolescent controls (*n* = 55), adult users (*n* = 67), and adult controls (*n* = 62). Error bars represent 95% confidence intervals. Both age-group and user-group main effects were non-significant, and the interaction was non-significant (ns)

In line with the stop signal task exclusion criteria (see supplementary materials), 16 participants were excluded. Additionally, there were 2 participants with missing data. After these participants were excluded, sample sizes were as follows: adolescent users *n* = 72; adolescent controls *n* = 55; adult users *n* = 67; adult controls *n* = 62 (Table 3). For the SSRT data, one extreme outlier in the adolescent user-group was found and Winsorized.

The interaction between age-group and user-group was non-significant (*F*(1,252) = 0.458, *p* = 0.499, η_p_^2^ = 0.002). The main effects of age-group (*F*(1,252) = 2.840, *p* = 0.093, η_p_^2^ = 0.011) and user-group (*F*(1,252) = 0.391, *p* = 0.532, η_p_^2^ = 0.002) were also non-significant. Inclusion of covariates in the ANCOVA did not change the pattern of these results (see supplementary table S2). Post hoc Bayesian analyses supported the null hypothesis of no difference between user-groups (BF_01_ = 8.006) and between adolescent users and adolescent controls (BF_01_ = 4.935). Mean SSRT across all groups can be seen in Fig. 3. For secondary variable results, see supplementary tables S9 and S10. In brief, although the users and controls had equivalent SSRTs, they had different profiles of responding. Users had slower go RTs and responded to go stimuli less accurately than controls, but users responded to stop stimuli more accurately than controls.

In users only, the interaction between cannabis frequency and age-group was non-significant (*F*(1,135) = 0.229, *p* = 0.633, η_p_^2^ = 0.002). The main effect of cannabis use frequency was also non-significant (*F*(1,135) = 0.100, *p* = 0.752, η_p_^2^ = 0.001). This pattern of results did not change with inclusion of covariates in the ANCOVA (see supplementary table S3).

### Age-of-onset exploratory analyses

Within adult users, there were no significant associations between the age cannabis was first used and VEM, SWM, or response inhibition (see supplementary materials).

## Discussion

This cross-sectional study investigated verbal episodic memory (VEM), spatial working memory (SWM) and response inhibition in adolescent cannabis users, adolescent controls, adult users, and adult controls. Users had worse VEM than controls. There was a trend-level interaction between user-group and age-group, in which adult users performed worse than adult controls, while this was not the case for adolescents. However, this trend interaction did not persist after adjusting for covariates, thus we conclude there is not good evidence for a differential relationship in adolescents and adults. User-group, age-group, and their interaction, were not associated with SWM or response inhibition. Indeed, Bayesian analyses supported users and controls having equivalent SWM and response inhibition capacity, across both age-groups and in adolescents only. Within users, there was tentative evidence that cannabis use frequency was negatively associated with VEM, but no evidence that cannabis use frequency was associated with SWM or response inhibition.

In the prose recall task, unadjusted analyses revealed that cannabis users recalled significantly less verbal information after a delay than controls, demonstrating poorer VEM. It is important to note that the effect size was small (η_p_^2^ = 0.020) and the overlap between the groups was large. This aligns with previous research (Broyd et al. 2016; Gonzalez et al. 2012; Schoeler et al. 2016; Solowij et al. 2011), demonstrating a small but significant deficit in cannabis users compared to controls on delayed recall. After adjusting for our pre-defined covariates, the main effect of user-group became only marginally significant (*p* = 0.053). This was likely due to some variance in delayed recall being accounted for by the covariates SES and WTAR (see supplementary table S3). However, the user-group main effect remains close to our alpha value and is consistent with most previous research.

Collectively, our results and the extant literature (Broyd et al. 2016; Schoeler et al. 2016) imply there is a weak but significant association between chronic cannabis use and impaired delayed recall. Consistent with our results, in the meta-analysis of 7,697 healthy participants (Schoeler et al. 2016) found a small (*d* = 0.39) association between chronic cannabis use and poorer verbal delayed recall. In our study, we found an effect size of η_p_^2^ = 0.020 (*d* = 0.28), with an absolute mean difference of 0.75 (12.8%) idea units. Since the effect size of the user vs. control difference in VEM is small, the clinical or educational relevance is dubious, as highlighted by past researchers (Scott et al. 2017). Norms for prose recall performance do not exist, so we cannot place our group means within a normal population distribution. However, for comparison, and to highlight the small size of the effect observed here, people with dementia were almost ten times worse than healthy controls on the prose recall task, with a Cohen’s d of four, and an absolute mean difference of six points (Greene et al. 1996). Furthermore, the small difference between cannabis users and controls may reverse upon prolonged abstinence (Pope et al. 2001; Schoeler et al. 2016; Scott et al. 2018).

We found a non-significant, but trend, interaction between age-group and user-group on VEM. In contrast to our hypothesis, adult users performed worse than adult controls, but adolescent users did not differ from adolescent controls. Not only was this a trend result, but the interaction was lost after inclusion of covariates. Hence, despite the unadjusted significant interaction, we conclude that adults do not have a stronger relationship between long-term cannabis use and VEM than adolescents. Previous studies have found that those with an earlier age of cannabis use onset recalled less words than those with a later age-of-onset (Becker et al. 2018; Solowij et al. 2011). Although our adolescent cannabis users had similar age-of-onsets and cannabis use frequency to Solowij and colleagues’ users, we found that adolescents were not more vulnerable to the effects of cannabis on VEM than adults.

In contrast to our hypothesis, in both unadjusted and adjusted analyses, we found no significant differences in SWM or response inhibition between cannabis users and controls. Bayesian analyses supported these null results, providing evidence that chronic cannabis use is not associated with either response inhibition or spatial working memory. Our results are consistent with much of the previous research, which has not found evidence for an association between cannabis use and response inhibition (Gonzalez et al. 2012; Grant et al. 2012; Hester et al. 2009; Tapert et al. 2007; Wallace et al. 2020) or SWM (Cousijn et al. 2014b; Desrosiers et al. 2015; Grant et al. 2012). In contrast, one large longitudinal cohort study found that, in some but not all analyses, cannabis use over a 4-year period was associated with impaired SWM in adolescents (Morin et al. 2019; Scott et al. 2017).

Chronic cannabis use may be associated with reduced VEM, but not SWM or response inhibition, due to neurobiological factors. Research has implicated the hippocampus as an important structure for verbal (Sass et al. 1990; Johnson et al. (2001) and episodic memory (Vargha-Khadem et al. 1997; Moscovitch et al. 2016). The hippocampus has a high density of cannabinoid-1 receptors (CB1Rs) (Herkernham et al. 1991; Moldrich & Wenger 2000; Tsou et al. 1997), thus implicating CB1Rs in declarative memory formation. On the other hand, SWM and response inhibition are executive functions and are thought to be more reliant on the prefrontal cortex (Blasi et al. 2006; Horn et al. 2003; Rae et al. 2015), which has a lower CB1R density than the hippocampus (Auclair et al. 2000; Tsou et al. 1997). Speculatively, any downregulation and desensitisation of CB1Rs with chronic cannabis use may have a weaker functional, behavioural effect on SWM and response inhibition. However, this hypothesis is not entirely supported by the mixed fMRI literature that has sometimes, but not always, demonstrated differences between cannabis users and controls in the neural correlates of working memory and response inhibition (Jager et al. 2006; Kanayama et al. 2004; Tapert et al. 2007).

In both the spatial n-back and stop signal task, we found no significant interactions between the age and user-groups. Moreover, the trend interaction on VEM was lost after adjusting for covariates. The equivalence of adolescent users and controls on SWM and response inhibition was supported by Bayesian analyses. Furthermore, we found no significant interactions between age-group and cannabis use frequency. Finally, our exploratory analyses within adult users showed null associations between age-of-onset and task performance. Therefore, our study does not provide any evidence that 16–17-year-old adolescents have an increased vulnerability to cannabis-related VEM, SWM, or response inhibition impairments in comparison to 26–29-year-old adults. Nor does it provide evidence that a younger age-of-onset is associated with poorer cognitive function. Indeed, the overall picture is comprehensively in favour of no heightened adolescent sensitivity.

These results are consistent with some age-of-onset studies, but not others. Crucially, in meta-analyses, null associations between age of cannabis use onset and cognitive function, including working memory and executive function, have been reported (Lovell et al. 2020; Scott et al. 2017). However, earlier age-of-onset has been associated with worse verbal memory in some studies (Becker et al. 2018; Solowij et al. 2011), but not others (Fontes et al. 2011). To reconcile these differences, further studies are required which: (a) longitudinally track adolescents’ cognition as they grow up, and (b) compare adolescent cannabis users with adult users who initiated cannabis use at the same time as the adolescents, and adult users who initiated cannabis use after adolescence.

### Strengths and limitations

Our sample (*n* = 274) is large in comparison to many previous similar studies that have investigated cognitive function in adolescent cannabis users. Furthermore, the novel approach of comparing cannabis-matched adolescents and adults, alongside age- and gender-matched controls, permitted a direct comparison and investigation of adolescent vulnerability to cannabis. Adult cannabis users had not used cannabis frequently before the age of 18 and our controls all had limited exposure to cannabis or tobacco, reducing unmeasured confounding differences with users. Abstinence from all drugs was verified using biological measurements. Moreover, we pre-registered our protocol and analyses, adjusted for relevant covariates, and conducted Bayesian tests to support null findings.

Given our participant recruitment strategy, our sample is not representative of the general UK population or UK cannabis users. However, this sampling methodology was required in order to recruit frequent cannabis users and matched controls, and optimise power. This is common in observational drug research (Becker et al. 2018; Jacobus et al. 2015; Morgan et al. 2012), given baseline levels of frequent drug use are low in the general population. While we assessed a range of cognitive domains that previous research implicated in cannabis harms, there are many other aspects of cognition which we did not explore, including decision-making, processing speed, attention set-shifting and motor function, some of which have been shown to be linked to long-term cannabis use (Figueiredo et al. 2020; Lovell et al. 2020; Scott et al. 2017), and further research is needed to unpack adolescent vulnerability in these areas. Although adolescent and adult users were well matched on cannabis use frequency and cannabis type, adolescents estimated using a significantly greater quantity of cannabis on a day of use and had greater problematic cannabis use than adults, while adult users had used cannabis regularly for a longer duration than adolescents. This was a cross-sectional study, therefore it cannot detect changes in the groups’ performance over time, when differences may emerge. Future research should also recruit younger cannabis users to test whether adolescent vulnerability appears at younger ages and ideally compare cannabis users against age-based population norms for cognitive function.

## Conclusions

This cross-sectional study found a significant, but small negative association between chronic cannabis use and VEM. There were no relationships between user-group and response inhibition or SWM. These results were supported by Bayesian analyses. We did not find evidence for an age-specific cannabis vulnerability for VEM, SWM, or response inhibition. These results do not lend support to the hypothesis that adolescents are at greater risk of cannabis-induced cognitive impairment. However, large longitudinal studies of cannabis-using and non-using adolescents and adults are needed to confirm this.

## Supplementary Information

Below is the link to the electronic supplementary material.

Supplementary file1 (DOCX 787 kb)
