# Correction to: The CannTeen study: verbal episodic memory, spatial working memory, and response inhibition in adolescent and adult cannabis users and age‑matched controls

**DOI:** 10.1007/s00213-022-06169-7

**Published:** 2022-06-01

**Authors:** W. Lawn, N. Fernandez‑Vinson, C. Mokrysz, G. Hogg, R. Lees, K. Trinci, K. Petrilli, A. Borissova, S. Ofori, S. Waters, P. Michór, M. B. Wall, T. P. Freeman, H. V. Curran

**Affiliations:** 1 Clinical Psychopharmacology Unit, University College London, London, UK; 2 Department of Addictions, Institute of Psychiatry, Psychology and Neuroscience, King’s College London, London, UK; 3 Department of Psychology, Institute of Psychiatry, Psychology and Neuroscience, King’s College London, London, UK; 4 Faculty of Biology, Medicine and Health, University of Manchester, Manchester, UK; 5 Addiction and Mental Health Group (AIM), Department of Psychology, University of Bath, Bath, UK; 6 Department of Neuroimaging, Institute of Psychiatry, Psychology and Neuroscience, King’s College London, London, UK; 7 NIHR University College London Hospitals Biomedical Research Centre, University College Hospital, London, UK; 8 Department of Psychiatry, University of Oxford, Warneford Hospital, Oxford, UK; 9 School of Life Sciences, University of Warwick, Coventry, UK; 10 Invicro London, Hammersmith Hospital, Burlington Danes Building, Du Cane Road, London, UK

**Correction to: Psychopharmacology**

In the published paper, it was found out that the incorrect supplementary materials were uploaded.

The original article has been corrected.

