# Supplementary material for: The CannTeen study: verbal episodic memory, spatial working memory, and response inhibition in adolescent and adult cannabis users and age-matched controls
Source: Psychopharmacology (Berl). 2022 Apr 29;239(5):1629–41. doi: 10.1007/s00213-022-06143-3 (PMC9110435; doi:10.1007/s00213-022-06143-3)
Supplement: Supplementary file 1 — Supplementary file1 (DOCX 787 kb) [file 213_2022_6143_MOESM1_ESM.docx]

**SUPPLEMENTARY MATERIALS**

**THE CANNTEEN STUDY: VERBAL EPISODIC MEMORY, SPATIAL WORKING MEMORY, AND RESPONSE INHIBITION IN ADOLESCENT AND ADULT CANNABIS USERS AND AGE-MATCHED CONTROLS**

**Authors**

W Lawn^1,2,3^, N Fernandez-Vinson^1,4^, C Mokrysz^1^, G Hogg^1^, R Lees^5^, K Trinci^1^, K Petrilli^5^, A Borissova^6,7^, S Ofori^1^, S Waters^8^, P Michór^9^, MB Wall^1,10^, TP Freeman^1,5^ & HV Curran^1^

**Affiliations**

1 Clinical Psychopharmacology Unit, University College London, London, United Kingdom. 2 Department of Addictions, Institute of Psychiatry, Psychology and Neuroscience, King’s College London, London, United Kingdom. 3 Department of Psychology, Institute of Psychiatry, Psychology and Neuroscience, King’s College London, London, United Kingdom 4 Faculty of Biology, Medicine and Health, University of Manchester, Manchester, United Kingdom 5 Addiction and Mental Health Group (AIM), Psychology Department, University of Bath, Bath, United Kingdom 6 Department of Neuroimaging, Institute of Psychiatry, Psychology and Neuroscience, King’s College London, London, United Kingdom 7 NIHR University College London Hospitals Biomedical Research Centre, University College Hospital, London, United Kingdom 8 Department of Psychiatry, University of Oxford, Warneford Hospital, Oxford, UK 9 School of Life Sciences, University of Warwick, Coventry, United Kingdom 10 Invicro London, Burlington Danes Building, Hammersmith Hospital, Du Cane Road, London, United Kingdom.

Table of Contents

[**Section 1: Supplementary introductory material** 2](#_Toc101692089)

[**Section 2: Eligibility Criteria** 3](#_Toc101692090)

[**Section 3: Measures** 5](#_Toc101692091)

[**Section 4: Statistical Analyses** 11](#_Toc101692092)

[**Section 5: Additional Results** 12](#_Toc101692093)

[**Section 6: Substance Use Variables** 25](#_Toc101692094)

[**Section 7: Supplementary Discussion** 27](#_Toc101692095)

# **Section 1: Supplementary introductory material**

*Verbal episodic memory*

Verbal episodic memory (VEM) develops throughout childhood, with some evidence suggesting maturation by 11 years (Vakil et al., 2010), and other research implying continued development until at least 16 years (Curry et al., 1986; Sowell et al., 2001). A systematic review concluded that verbal memory is one of the cognitive processes most consistently affected by cannabis use (Broyd et al., 2016). Longitudinal studies in adolescents have shown both overall, between-subjects differences between users and controls after cannabis abstinence of 3 weeks (Jacobus et al., 2015) and within-subjects prediction of worsening immediate verbal recall (Duperrouzel et al., 2019).

*Spatial working memory*

Spatial working memory (SWM) precision increases with age (Schutte et al., 2003; Schutte & Spencer, 2009), with lifetime SWM development following an inverted-U shape pattern, where young adults have better functioning than children and older adults (Cowan et al., 2006).

In one smaller longitudinal study, poorer SWM behavioural performance and SWM neural correlates predicted future cannabis use, but not vice versa (Tervo-Clemmens et al., 2018). Other studies have found that after 12 or more hours abstinence, adolescent cannabis users had significantly worse SWM than age-matched non-using controls (Harvey et al., 2007). However, Becker and colleagues reported that, although adults with adolescent onset cannabis use had poorer working memory than non-using controls, age of onset did not moderate this effect within users (Becker et al., 2018).

*Response inhibition*

Response inhibition improves throughout childhood (Booth et al., 2003; Tamm et al., 2002), reaching full maturation around mid-to-late adolescence (Luna et al., 2009).

# **Section 2: Eligibility Criteria**

**Table S1**. Full inclusion and exclusion criteria

|  | Inclusion Criteria | Exclusion Criteria |
| --- | --- | --- |
| All participants | • Able to come to UCL five times over the next year  • Capacity to give informed consent  • Normal or corrected-to normal vision  • Fluent in English | • Any illicit drug use within 48 hours of the behavioural baseline session, verified with self-report and saliva testing  • Any cannabis or alcohol use within 12 hours of the behavioural baseline session, verified with self-report and saliva/breathalyser testing.  • Personal history of a diagnosed psychotic episode or disorder  • Any one illicit drug taken >2 days/month (averaged over last 3 months) (except laughing gas)  • Use of laughing gas >1 day/week (averaged over last 3 months)  • Receiving treatment for any mental health condition, including cannabis dependence, in the last month  • Unwilling to give blood samples or likely to faint on blood sampling  • Current daily use of a medication which is commonly psychotropic  • Any mental or physical health problem judged to be problematic for the study, by a medical doctor |
| Teenage cannabis users | • Aged 16-17 years  • Cannabis use at a frequency of 1-7 days/week (averaged over last 3 months) | • Age-adjusted BMI <2^nd^ percentile or >99.6^th^ percentile |
| Teenage controls | • Aged 16-17 years  • Between 1 and 10 days of lifetime cannabis use or 0 days of lifetime cannabis use and at least 1 day of lifetime cigarette/roll-up use | • Age-adjusted BMI <2^nd^ percentile or >99.6^th^ percentile  • Cannabis use more than once in the last 3 months before behavioural baseline session  • Cannabis use in the month prior to the behavioural baseline session |
| Adult cannabis users | • Aged 26-29 years  • Cannabis use at a frequency of 1-7 days/week (averaged over last 3 months) | • Before the age of 18, cannabis use at a frequency of once per week or more for a period of 3 months or more.  • BMI <18.5 or >34.9 |
| Adult controls | • Aged 26-29 years  • Between 1 and 10 days of lifetime cannabis use or 0 days of lifetime cannabis use and at least 1 day of lifetime cigarette/roll-up use | • Cannabis use more than once in the last 3 months before behavioural baseline session  • Cannabis use in the month prior to the baseline session  • BMI <18.5 or >34.9 |

# **Section 3: Measures**

**Stop signal task (figure S1)**:

Participants responded to white arrows as they appeared sequentially on the laptop screen (figure S1a). Participants were instructed to press the right arrow key with their right index finger if the arrow was pointing to the right, and the left arrow key with their left index finger if it was pointing to the left (Lawn et al., 2020). 25% of trials were stop trials, where the white arrow turns blue after a variable delay (figure S1b). Participants were told to inhibit their response if the arrow turned blue. Participants were instructed to respond to the white arrows as quickly and accurately as possible, and not wait for the arrow to turn blue. Each trial lasted 1500ms, with 500ms inter trial intervals. There were 200 trials in total, and therefore 50 stop trials.

Staircase tracking was used to adjust the stop signal delay (SSD, delay between go stimulus onset – white arrow - and stop signal – arrow turns blue) for each stop trial. Initially, the SSD was 250ms. When the participant successfully inhibited their response, the SSD was increased by 50ms for the next stop trial. When the participant was unsuccessful in inhibiting their response, SSD was decreased by 50ms. This was designed to result in each participant having a 50% chance of successful response inhibition, so that a reliable stop signal reaction time (SSRT) can be calculated (Verbruggen et al., 2019a; Verbruggen & Logan, 2008).

The primary outcome variable for the stop signal task was SSRT, an estimation of the latency of the stop process (Verbruggen et al., 2019b; Verbruggen & Logan, 2008). SSRT is calculated according to the mean method, where SSRT = mean RT on go trials – mean SSD (Matzke et al., 2018). Secondary outcome variables were mean SSD, mean go RT, go trial % correct and stop trial % correct. Participants were excluded if: their probability of responding to a stop signal was lower than 25% or higher than 75%, percentage of correct go responses was below 80%, percentage of go errors was higher than 10% or if their SSRT was below 50ms. Go trials were deemed successful even if the opposite button was pressed relative to the arrow that was presented. However, as stated, participants were excluded if this happened >10% of the time; this only happened once. For those included, 18 participants had >4 trials in which they pressed the incorrect button direction.

**Figure S1.** The stop-signal task. (a) Example of a go trial. A white arrow appears on the screen, without turning blue. Participants must press either the left or right arrow key, depending on which way the arrow is pointing. (b) Example of a stop trial. The white arrow appears on the screen, which then turns blue after a variable delay. Participants are instructed to inhibit their response and not press any key (Lawn et al., 2020).


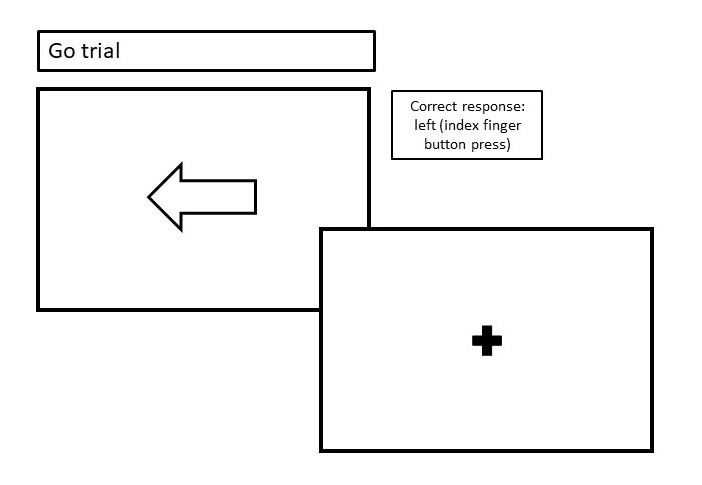
(a)

b)


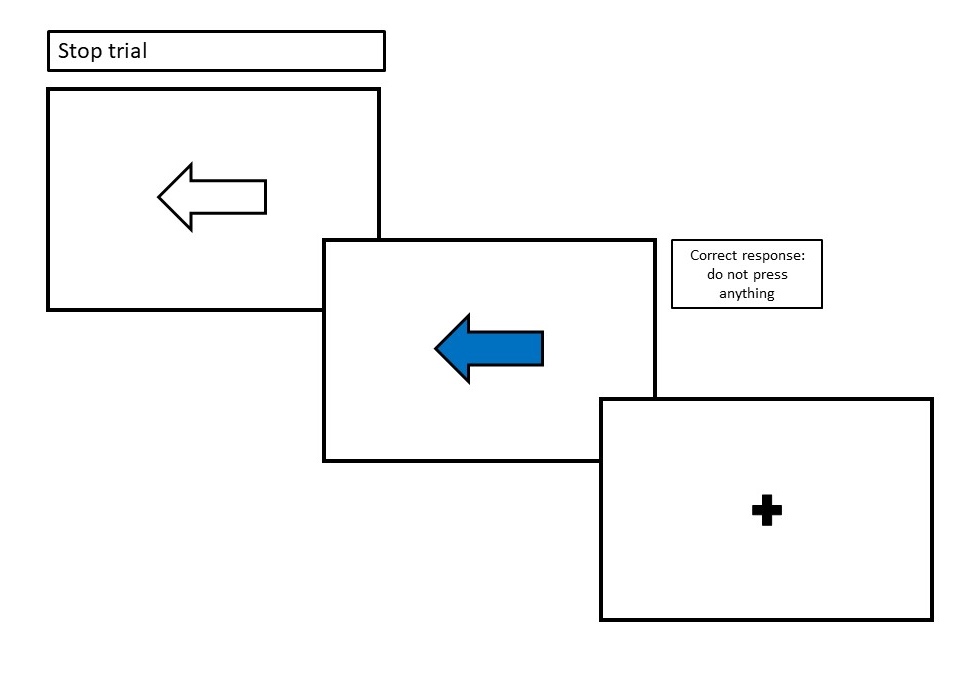


**Spatial n-back task (figure S2)**:

First, a blue square was presented to the participant at one of six positions on the screen around a central fixation point for 600ms. There was a pause of 1500ms before the next square. One trial lasts 2100ms in total. Responses were only recorded if made before the end of each trial. This task had four conditions: 0-back, 1-back, 2-back and 3-back, appearing in this order. New instructions appeared before each condition. Each condition contained 20 ‘yes’ and 20 ‘no’ trials. The running task time is 5 minutes and 36 seconds. Participants were reminded to respond as quickly and accurately as possible.

In the 0-back condition, the participant must determine if the square is in the ‘12 o’clock’ position (by pressing the left index finger button indicating ‘yes’) or in one of the other five positions (by pressing the right index finger button indicating ‘no’). In the 1-back condition, the participant must determine if the square appears in the same position as the square presented one trial before (‘yes’ trial, target) or in a different position (‘no’ trial, non-target). In the 2-back condition, the participant must determine if the square is in the same position as the square presented two trials before it (‘yes’ trial, target) or in a different position (‘no’ trial, non-target). In the 3-back condition, the participant must determine if the square is in the same position as the square presented three trials before it (‘yes’ trial, target) or in a different position (‘no’ trial, non-target)

We calculated performance at each load: 0-back, 1-back, 2-back and 3-back. The most sensitive and specific outcome measure of n-back performance is discriminability (d’) (Haatveit et al., 2010). This is calculated by: d′ = Z_Proportion_ _of Hits_–Z_Proportions_ _of False Alarm_. Proportion of hits = number of successful hits/number of targets. Proportion of false alarms = number of false alarms/number of non-targets.

If proportions of hits is equal to 1, this is replaced by 1-1/(2*number of targets). In this task, 1 is therefore replaced by 1 –1/40 = 0.975. If the proportion of false alarms is equal to 0, this is replaced by1/(2*number of non-targets). In this task 0 is therefore replaced by 1/40 = 0.025. We analysed performance at each load separately, however the primary outcome variable was d’ in the 3-back condition. Participants who did not successfully complete the task, who either reacted in under 150ms on ≥5 trials in any one condition, or who ‘missed’ ≥5 trials, were excluded from statistical analyses. Secondary outcome variables were d’ in the 0-back, 1-back and 2-back conditions, as well as RT and % trials correct for all conditions.

**Figure S2.** Spatial n-back task. A) 0-back; Participant must determine if the square is in the ‘12 o’clock’ position (by pressing ‘yes’ button on keyboard) or elsewhere (by pressing ‘no’). B) 1-back; Participant must determine if the square appears in the same position as the square presented one trial before (‘yes’ trial, target) or elsewhere (‘no’ trial, non-target). C) 2-back; Participant must determine if the square is in the same position as the square presented two trials before it (‘yes’ trial, target) or elsewhere (‘no’ trial, non-target). D) 3-back; Participant must determine if the square is in the same position as the square presented three trials before it (‘yes’ trial, target) or elsewhere (‘no’ trial, non-target) (Lawn et al., 2020)**.**

d)

c)

a)

b)


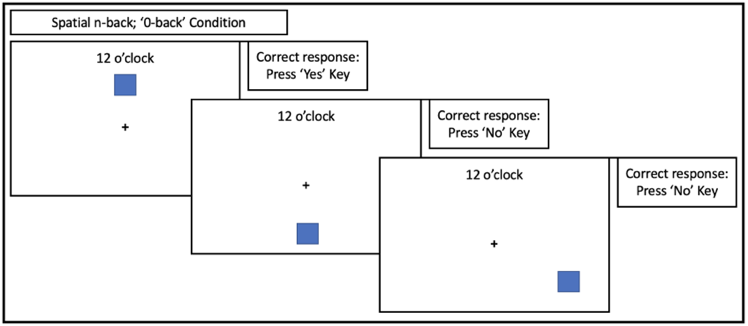

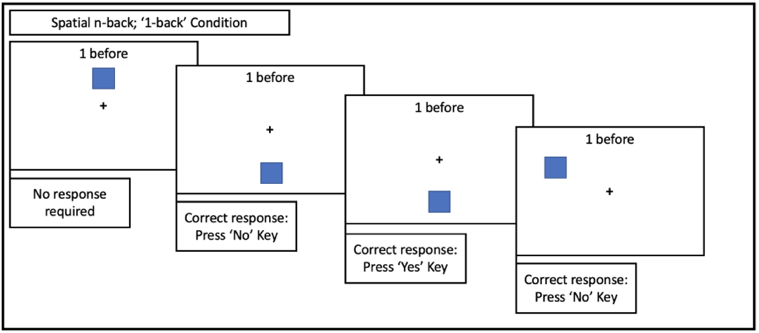

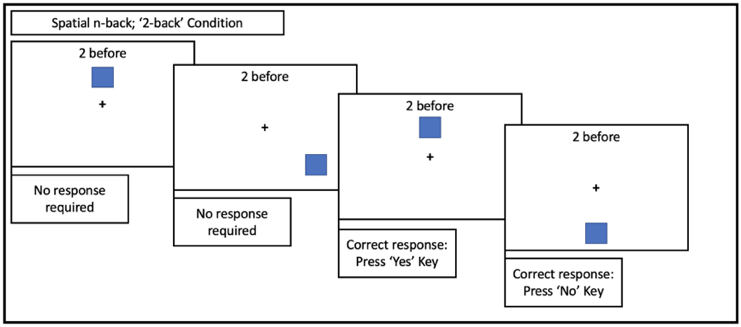

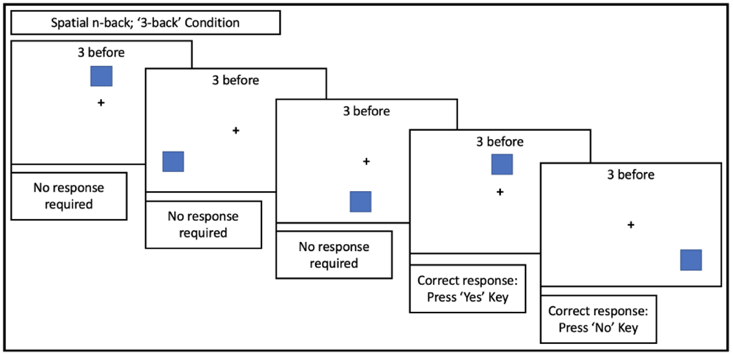


**Gender**: We asked participants what their gender was. Participants could respond ‘male’, ‘female’, ‘other’, or ‘prefer not to say’. Participants only responded male or female. Female was coded 0 and male was coded 1.

**Substance use frequency:** Cannabis, alcohol, tobacco and illicit drug use frequency were all assessed using the TLFB method (Robinson et al., 2014). Participants stated which type of cannabis they used. Cannabis was categorized into strong herbal cannabis (i.e. high-strength, ‘skunk’, ‘high-grade’, sinsemilla, seedless, indoor-grown); weak herbal (i.e. low-strength herbal, outdoor-grown seeded cannabis, ‘Thaiweed’, ‘brickweed’, ‘ditchweed’); and hash (i.e. resin, the brown solid created by pressing resin glands/trichomes) (Freeman & Winstock, 2015). Participants were prompted to recall the days on which they used these drugs in the 12 weeks prior to baseline. An average use frequency for each drug was calculated in days/week.

For pre-defined covariates, participants were categorized into the following groups: Alcohol use≥2 days/week (coded 1) or Alcohol use<2 days/week (coded 0); daily cigarette or roll-up tobacco use (≥ 6.5 days/week) (coded 1) or non-daily use (coded 0); and illicit drug use≥1 day/month (coded 1) or illicit drug use<1 day/month (coded 0).

**Risk-Taking 18 (RT18):** An 18-item questionnaire assessing risk-taking behaviour. Total scores range from 0-18, with higher scores indicating higher levels of risk taking behaviour (De Haan et al., 2011)

**Socio-economic status (SES):** Assessed using participants’ mother’s educational level. Participants are categorized into those whose mothers have an educational level of undergraduate degree or above (coded 1), and those whose mothers have an educational level below undergraduate degree (coded 0).

**Premorbid verbal intelligence:** Assessed using the Wechsler test of adult reading (WTAR) (Holdnack, 2001). Participants read a list of 50 words out loud and were marked on correct pronunciation. Scores were age adjusted scores and standardized to a normal IQ scale, with mean 100. Scores below 85 were replaced with the value 85.

**Alcohol Use Disorder Identification Test (AUDIT):** A 10-item questionnaire assessing hazardous drinking and dependence. Scores range from 0-40 (Bohn & Babor, 1995).

**Cannabis Use Disorder Identification Test- Revised (CUDIT-R):** An 8-item questionnaire assessing hazardous cannabis use and dependence. Scores range from 0-32 (Adamson et al., 2010)

**Delta-9-tetrahydrocannabinol (THC) concentration:** Cannabis users were asked if they were willing to donate a small amount (roughly 0.3g) of their usual cannabis to us. The cannabis was then analysed for THC concentration using ultra high performance liquid chromatography (UPLC).

**Breathalyzer Test:** Participants blood alcohol levels were assessed using a Lion Alcometer 500 breathalyzer at baseline, to check alcohol abstinence. Only those with a blood alcohol of 0 completed the session.

**Saliva drug tests:** Instant saliva drugs tests (either Alere DDSV 703 or ALLTEST DSD-867MET/C) were administered at baseline, assessing THC, opiates, benzodiazepine, methamphetamine, amphetamine and opiates in saliva. Only those with negative saliva tests for all drugs completed the session.

# **Section 4: Statistical Analyses**

***Power***

The project was not powered specifically for this analysis. The project was powered to detect a cross-sectional group difference in cannabis use disorder between adolescent and adult cannabis users (Lawn et al., 2020), with Cohen’s d=0.6, ɑ=0.05, power=0.95, requiring 148 users. We in fact recruited 147 users and therefore recruited a similar number of controls. Crucially, in terms of detecting age-group by user-group interactions in this baseline data (n=274), with an assumed power of 0.95 we are powered to detect small to medium interactions of size f≥0.22, and with an assumed power of 0.8 we are powered to detect small to medium interactions of size f≥0.17.

***Primary and secondary analyses***

For the stop signal task, the primary outcome variable was SSRT. Secondary outcome variables were mean SSD, mean go RT, stop trial % correct and go trial % correct.

For the prose recall task, the primary outcome variable was the number of idea units recalled at the delayed time point. The secondary outcome variable was the number of idea units recalled at the immediate time point.

For n-back, the primary outcome variable was 3-back d’. The secondary outcome variables were 0-back, 1-back and 2-back d’, RT and % trials correct, in addition to 3-back RT and % trials correct.

***Statistical Assumptions***

For outcome variables, extreme outliers (lying more than 3 times the interquartile range away from the lower or upper quartile) were Winsorized with the nearest non-outlying datapoint. For ANOVAs and ANCOVAs, Levene’s test of equality of variances was checked, and found to be non-significant unless otherwise stated. Homogeneity of variance was also checked by inspecting graphs of Predicted (x) vs Residual (y) values. Variance was found to be homogenous, unless otherwise stated. Normality of residuals were checked using Kolmogorov-Smirnov tests and visual inspection of histograms, and residuals were found to have a normal distribution unless otherwise stated. Maximum Cook’s distances were calculated and found to be small (<1), with the majority being <0.3. For ANCOVAs, multicollinearity was checked by running correlations between predictors. Unless otherwise stated, correlations between predictors were r < 0.45, indicating that multicollinearity was not an issue. An interim analysis was conducted before all data were collected, when C. Mokrysz presented data at a conference. Hypotheses remained the same throughout and data analysis was not affected.

# **Section 5: Additional Results**

*Participant characteristics (see Tables 1 and 2)*

All groups had a similar number of males and females. Adolescent users (17.1 years) and adolescent controls (17.1 years) were matched on age (t_137_=0.224, p=0.823, d=0.038), as were adult users (27.6 years) and adult controls (27.4 years) (t_145_=1.232, p=0.220, d=0.212).

Adolescent users (3.7 days/week) and adult users (4.1 days/week) were matched on cannabis use frequency (t_145_=1.198, p=0.233, d=0.198). The time since last cannabis use was similar for adolescent users (2.4 days, SD=2.6, range: 0.50-14.0 days) and adult users (2.5 days, SD=4.6, range: 0.5-35.0) (t_145_=0.118, p=0.906, d=0.019). A similar number of adolescent users (n=69, 90.8%) and adult users (n=59, 83.1%) used strong herbal cannabis as their most common type of cannabis, and these distributions did not differ (χ_3_^2^=3.866, p=0.276) (table S14). Furthermore, albeit in a small subsample, the adolescent users (21.1%, SD=5.2, n=14) and adult users (21.3%, SD=4.6, n=12), used strong cannabis of a similar THC concentration (t_24_=0.100, p=0.921, d=0.041).

Significantly more adolescents were in the high SES category than adults (𝜒^2^_1_=5.345, p=0.021), they used alcohol less frequently than adults (F_1,270_=44.114, p<0.001, η_p_^2^=0.140), and they had higher RT-18 scores than adults (F_1,270_=21.077, p<0.001, η_p_^2^=0.072).

Users had higher AUDIT scores than controls (F_1,270_=7.099, p=0.008, η_p_^2^=0.026), they were more likely to use tobacco on a daily basis than controls (𝜒^2^_1_=8.467, p=0.004), they had higher RT-18 scores than controls (F_1,270_=14.847, p<0.001, η_p_^2^=0.052), and were more likely to use other illicit drugs on a monthly basis than controls (𝜒^2^_1_=61.103, p<0.001). Additionally, adolescent users were more likely to use another illicit drug on a monthly basis than adult users (𝜒^2^_1_=17.183, p<0.001). However, the frequency of other illicit drug use was infrequent in those, when considering those who had used in the past three months, and the frequency did not differ between groups (adolescent user: 1.83 days/month; adolescent control: 1.17 days/month; adult user: 1.05 days/month; adult control: 0.55 days/month).

For controls, adults had used cannabis slightly more times (4.5) in their life than adolescents (3.4) (t_125_=2.067, p=0.041, d=0.367) (table 2).

For users, adolescents (14.6 years) first tried cannabis at an earlier age than adults (18.5 years) (t_144_=9.647, p<0.001, d=1.598) and they reported using more cannabis (1.1g) on a day of use than adults (0.6g) (t_142_=3.623, p<0.001, d=0.605). Adult users (5.3 years) had longer durations of weekly cannabis use than adolescent users (1.5 years) (t_145_=11.577, p<0.001, d=1.911)

**Table S2**: Full ANCOVA results, with pre-defined covariates, for primary outcome variables SSRT, 3-back d’ and delayed recall. RT-18 = risk-taking 18; Alcohol = twice-weekly alcohol use; tobacco = daily cigarette or roll-up use; other illicit drugs = monthly use of any other illicit drug; SES = socioeconomic status (maternal education); WTAR = Wechsler test of adult reading.

|  | **F** | **df** | **p** | **η_p_^2^** |
| --- | --- | --- | --- | --- |
| **Prose recall – delayed recall score** |  |  |  |  |
| User-Group | 3.780 | 1,252 | .053 | .015 |
| Age-Group | 9.351 | 1,252 | .002 | .036 |
| User-Group*Age-Group | 1.547 | 1,252 | .215 | .006 |
| Gender | 1.611 | 1,252 | .206 | .006 |
| RT-18 | 0.142 | 1,252 | .706 | .001 |
| Alcohol | 0.994 | 1,252 | .320 | .004 |
| Tobacco | 0.754 | 1,252 | .386 | .003 |
| Other illicit drugs | 0.468 | 1,252 | .495 | .002 |
| SES | 10.747 | 1,252 | .001 | .041 |
| WTAR | 13.378 | 1,252 | <.001 | .050 |
| **Spatial n-back – d’ in the 3-back** |  |  |  |  |
| User-Group | 0.938 | 1,219 | .334 | .004 |
| Age-Group | 0.992 | 1,219 | .338 | .004 |
| User-Group*Age-Group | 1.361 | 1,219 | .245 | .006 |
| Gender | 13.191 | 1,219 | <.001 | .057 |
| RT-18 | 3.249 | 1,219 | .073 | .015 |
| Alcohol | 7.879 | 1,219 | .005 | .035 |
| Tobacco | 0.204 | 1,219 | .652 | .001 |
| Other illicit drugs | 2.144 | 1,219 | .145 | .010 |
| SES | 0.522 | 1,219 | .471 | .002 |
| WTAR | 3.950 | 1,219 | .048 | .018 |
| **Stop Signal Task- SSRT** |  |  |  |  |
| User-Group | 0.410 | 1,237 | .523 | .002 |
| Age-Group | 2.782 | 1,237 | .097 | .012 |
| User-Group*Age-Group | 0.398 | 1,237 | .529 | .002 |
| Gender | 0.024 | 1,237 | .878 | <.001 |
| RT-18 | 1.854 | 1,237 | .175 | .008 |
| Alcohol | 0.554 | 1,237 | .457 | .002 |
| Tobacco | 4.278 | 1,237 | .040 | .018 |
| Other illicit drugs | 0.104 | 1,237 | .747 | <.001 |
| SES | 0.070 | 1,237 | .792 | <.001 |
| WTAR | 8.671 | 1,237 | .004 | .035 |

**Table S3:** Full results from ANCOVA analyses only in users, with cannabis use frequency in days/week as a covariate. RT-18 = risk-taking 18; Alcohol = twice-weekly alcohol use; tobacco = daily cigarette or roll-up use; other illicit drugs = monthly use of any other illicit drug; SES = socioeconomic status (maternal education); WTAR = Wechsler test of adult reading.

|  | **F** | **df** | **p** | **η_p_^2^** |
| --- | --- | --- | --- | --- |
| **Prose recall – delayed recall score** |  |  |  |  |
| Age-Group | 2.174 | 1,127 | .143 | .017 |
| Cannabis frequency | 3.705 | 1,127 | .056 | .028 |
| Age-Group*Cannabis Frequency | 0.400 | 1,127 | .528 | .003 |
| Gender | 4.745 | 1,127 | .031 | .036 |
| RT-18 | 0.016 | 1,127 | .900 | <.001 |
| Alcohol | 0.098 | 1,127 | .755 | .001 |
| Tobacco | 0.010 | 1,127 | .922 | <.001 |
| Other illicit drugs | 0.902 | 1,127 | .344 | .007 |
| SES | 7.224 | 1,127 | .008 | .054 |
| WTAR | 4.272 | 1,127 | .041 | .033 |
| **Spatial n-back – d’ in the 3-back** |  |  |  |  |
| Age-Group | 0.140 | 1,104 | .709 | .001 |
| Cannabis frequency | 1.025 | 1,104 | .314 | .010 |
| Age-Group*Cannabis frequency | 0.027 | 1,104 | .870 | <.001 |
| Gender | 9.845 | 1,104 | .002 | .086 |
| RT-18 | 7.890 | 1,104 | .006 | .071 |
| Alcohol | 0.090 | 1,104 | .764 | .001 |
| Tobacco | 0.017 | 1,104 | .896 | <.001 |
| Other illicit drugs | 0.527 | 1,104 | .470 | .005 |
| SES | 0.075 | 1,104 | .784 | .001 |
| WTAR | 3.058 | 1,104 | .083 | .029 |
| **Stop Signal Task- SSRT** |  |  |  |  |
| Age-Group | 0.093 | 1,121 | .761 | .001 |
| Cannabis frequency | 0.001 | 1,121 | .978 | <.001 |
| Age-Group*Cannabis frequency | 0.155 | 1,121 | .694 | .001 |
| Gender | 0.092 | 1,121 | .762 | .001 |
| RT-18 | 4.289 | 1,121 | .040 | .034 |
| Alcohol | 1.562 | 1,121 | .214 | .013 |
| Tobacco | 3.804 | 1,121 | .053 | .030 |
| Other illicit drugs | 0.771 | 1,121 | .382 | .006 |
| SES | 0.124 | 1,121 | .725 | .001 |
| WTAR | 0.810 | 1,121 | .370 | .007 |

*Immediate Recall Results*

**Table S4.** Mean immediate recall scores across the four groups. 95% confidence intervals are shown in brackets.

|  | **Adolescent**  **User** | **Adolescent Control** | **Adult**  **User** | **Adult**  **Control** |
| --- | --- | --- | --- | --- |
| **Immediate Recall** | 5.618 [4.964-6.273] | 5.889 [5.355-6.423] | 6.109 [5.507-6.711] | 7.508 [6.762-8.254] |

**Table S5**. Full results from ANOVA analysis of immediate recall in the prose recall task, *p<0.05, **p<0.01, ***p<0.001.

|  | **F** | **df** | **p** | **η_p_^2^** | **Group Differences** |
| --- | --- | --- | --- | --- | --- |
| **Immediate Recall** |  |  |  |  |  |
| User-Group | 6.704 | 1,268 | .010 | .024 | Controls>Users* |
| Age-Group | 10.700 | 1,268 | .001 | .038 | Adults>Adolescents** |
| User-Group*Age-Group | 3.064 | 1,268 | .081 | .011 |  |

In line with the delayed recall, exploration of the trend User-Group*Age-Group interaction for prose recall (immediate) showed that within adults, users performed worse than controls (t(131)= 2.911, p=0.003, MD = 1.339), but within adolescents the difference was non-significant (t(137)=0.597, p=0.550, MD=0.270).

*Spatial n-back secondary outcome variable results*

**Table S6.** Mean values for all spatial n-back secondary outcome variables across the four groups. 95% confidence intervals are shown in brackets.

|  | **Adolescent**  **User** | | **Adolescent Control** | **Adult**  **User** | **Adult**  **Control** |
| --- | --- | --- | --- | --- | --- |
| **3-back RT** | | .823 [.769-.877] | .772 [.725-.819] | .841 [.793-.889] | .887 [.820-.935] |
| **3-back % Trial Correct** | 70.7 [67.4-74.0] | | 70.6 [67.5-73.6] | 70.3 [67.1-70.3] | 72.4 [68.7-76.1] |
| **2-back d’** | 2.022 [1.808-2.235] | | 2.063 [1.819-2.307] | 2.256 [2.014-2.497] | 2.248[1.948-2.547] |
| **2-back RT** | .813[.759-866] | | .772 [.719-.825] | .819 [.770-.868] | .815 [.752-.878] |
| **2-back % Trial Correct** | 80.3 [77.4-83.2] | | 80.4 [77.1-83.7] | 83.3 [80.2-86.4] | 82.2 [78.4-86.0] |
| **1-back d’** | 3.076 [2.935-3.217] | | 2.985 [2.830-3.140] | 3.187 [3.045-3.330] | 3.187 [3.028-3.344] |
| **1-back RT** | .621 [.585-.658] | | .580 [.550-.611] | .630 [.590-.670] | .645 [.606-.685] |
| **1-back % Trial Correct** | 92.5 [91.1-94.0] | | 92.2 [90.8-93.6] | 93.7 [92.4-95.0] | 93.5 [92.0-95.1] |
| **0-back d’** | 3.641 [3.553-3.749] | | 3.603 [3.491-3.714] | 3.669 [3.588-3.750] | 3.552 [3.431-3.673] |
| **0-back RT** | .483 [.459-.507] | | .466 [.445-.488] | .477 [.457-.497] | .485 [.466-.506] |
| **0-back % Trial Correct** | 97.7 [96.9-98.5] | | 97.3 [96.3-98.3] | 98.0 [97.3-98.6] | 96.9 [95.8-98.0] |

**Table S7.** Full ANOVA results of spatial n-back task secondary outcome variables. *p<0.05, **p<0.01, ***p<0.001.

|  | **F** | **df** | **p** | **η_p_^2^** | **Group Differences** |
| --- | --- | --- | --- | --- | --- |
| **3-back RT** |  |  |  |  |  |
| User-Group | 0.082 | 1,233 | .775 | <.001 | ns |
| Age-Group | 5.682 | 1,233 | .018 | .024 | Adolescents<Adults* |
| User-Group*Age-Group | 2.894 | 1,233 | .090 | .012 | ns |
| **3-back % Trials Correct** |  |  |  |  |  |
| User-Group | 0.355 | 1,233 | .552 | .002 | ns |
| Age-Group | 0.176 | 1,233 | .675 | .001 | ns |
| User-Group*Age-Group | 0.458 | 1,233 | .499 | .002 | ns |
| **2-back d’** |  |  |  |  |  |
| User-Group | 0.017 | 1,233 | .896 | <.001 | ns |
| Age-Group | 2.802 | 1,233 | .096 | .012 | ns |
| User-Group*Age-Group | 0.039 | 1,233 | .843 | <.001 | ns |
| **2-back RT** |  |  |  |  |  |
| User-Group | 0.663 | 1,233 | .416 | .003 | ns |
| Age-Group | 0.835 | 1,233 | .362 | .004 | ns |
| User-Group*Age-Group | 0.444 | 1,233 | .506 | .002 | ns |
| **2-back % Trials Correct** |  |  |  |  |  |
| User-Group | 0.105 | 1,233 | .747 | <.001 | ns |
| Age-Group | 2.073 | 1,233 | .151 | .009 | ns |
| User-Group*Age-Group | 0.148 | 1,233 | .700 | .001 | ns |
| **1-back d’** |  |  |  |  |  |
| User-Group | 0.337 | 1,233 | .540 | .002 | ns |
| Age-Group | 4.385 | 1,233 | .037 | .018 | Adolescents<Adults* |
| User-Group*Age-Group | 0.361 | 1,233 | .549 | .002 | ns |
| **1-back RT** |  |  |  |  |  |
| User-Group | 0.498 | 1,233 | .481 | .002 | ns |
| Age-Group | 4.058 | 1,233 | .045 | .017 | Adolescents<Adults* |
| User-Group*Age-Group | 2.389 | 1,233 | .124 | .010 | ns |
| **1-back % Trials Correct** |  |  |  |  |  |
| User-Group | 0.149 | 1,233 | .700 | .001 | ns |
| Age-Group | 3.149 | 1,233 | .077 | .013 | ns |
| User-Group*Age-Group | 0.021 | 1,233 | .885 | <.001 | ns |
|  | F | df | p | **η_p_^2^** | **Group Differences** |
| **0-back d’** |  |  |  |  |  |
| User-Group | 2.516 | 1,233 | .114 | .011 | ns |
| Age-Group | 0.101 | 1,233 | .751 | <.001 | ns |
| User-Group*Age-Group | 0.432 | 1,233 | .512 | .002 | ns |
| **0-back RT** |  |  |  |  |  |
| User-Group | 0.136 | 1,233 | .712 | .001 | ns |
| Age-Group | 0.422 | 1,233 | .516 | .002 | ns |
| User-Group*Age-Group | 1.354 | 1,233 | .246 | .006 | ns |
| **0-back % Trials Correct** |  |  |  |  |  |
| User-Group | 3.053 | 1,233 | .082 | .013 | ns |
| Age-Group | 0.050 | 1,233 | .822 | <.001 | ns |
| User-Group*Age-Group | 0.548 | 1,233 | .460 | .002 | ns |

**Table S8.** 1-back and 0-back Mann Whitney-U. For 1-back and 0-back data, assumptions were violated. Therefore, we conducted a set of non-parametric Mann Whitney-U tests between adult users and adult controls, and adolescent users and adolescent controls to further examine these results.

|  | Adolescents  Users vs. Controls | Adults  Users vs. Controls | |
| --- | --- | --- | --- |
| **1-back d’** |  |  | |
| U | 1691.500 | 1652 | |
| *p* | 0.473 | 0.867 | |
| **1-back RT** |  |  |  |
| U | 1560 | 1485 | |
| *p* | 0.163 | 0.277 | |
| **1-back % Trials Correct** |  |  | |
| U | 1710.500 | 1656 | |
| *p* | 0.534 | 0.884 | |
| **0-back d’** |  |  | |
| U | 1686 | 1475 | |
| *p* | 0.422 | 0.222 | |
| **0-back RT** |  |  | |
| U | 1645 | 1485 | |
| *p* | 0.340 | 0.277 | |
| **0-back % Trials Correct** |  |  | |
| U | 1681 | 1463 | |
| *p* | 0.409 | 0.198 | |

*Stop signal secondary outcome variable results*

For stop trial % correct, 6 extreme outliers were found and Winsorized. The extreme outliers were 1 adolescent user, 1 adolescent control and 4 adult controls. For go trial % correct, 8 extreme outliers were found and Winsorized. The extreme outliers were 1 adolescent user, 1 adolescent control, 4 adult users and 2 adult controls.

**Table S9**. Mean values for all stop signal secondary outcome variables across the four groups. 95% confidence intervals are shown in brackets.

|  | **Adolescent**  **User** | **Adolescent Control** | **Adult**  **User** | **Adult**  **Control** |
| --- | --- | --- | --- | --- |
| **Mean SSD** | .302 [.265-.338] | .257 [.215-.299] | .321 [.283-.359] | .269 [.229-.308] |
| **Mean Go RT (s)** | .567 [.534-.601] | .513 [.475-.552] | .572 [.537-.607] | .520 [.484-.556] |
| **Go Trial % Correct** | .979 [.974-.984] | .992 [.986-.998] | .984 [.979-.989] | .995 [.990-1.001 |
| **Stop Trial % Correct** | .504 [.492-.516] | .495 [4.81-.509] | .521 [.508-.534] | .498 [.485-.512] |

**Table S10**. Full results from ANOVA analyses of stop signal secondary variables. *p<0.05, **p<0.01, ***p<0.001, ns = non-significant.

|  | **F** | **df** | **p** | **η_p_^2^** | **Group Differences** |
| --- | --- | --- | --- | --- | --- |
| **Mean SSD** |  |  |  |  |  |
| User-Group | 5.898 | 1,252 | .016 | .023 | Controls<Users* |
| Age-Group | 0.609 | 1,252 | .436 | .002 | ns |
| User-Group*Age-Group | 0.037 | 1,252 | .847 | <.001 | ns |
| **Mean Go RT** |  |  |  |  |  |
| User-Group | 8.537 | 1,252 | .004 | .033 | Controls<Users** |
| Age-Group | 0.100 | 1,252 | .752 | <.001 | ns |
| User-Group*Age-Group | 0.002 | 1,252 | .996 | <.001 | ns |
| **Go Trial % Correct** |  |  |  |  |  |
| User-Group | 19.501 | 1,252 | <.001 | .072 | Controls>Users*** |
| Age-Group | 2.203 | 1,252 | .139 | .009 | ns |
| User-Group*Age-Group | 0.041 | 1,252 | .840 | <.000 | ns |
| **Stop Trial % Correct** |  |  |  |  |  |
| User-Group | 5.679 | 1,252 | .018 | .022 | Controls<Users* |
| Age-Group | 2.292 | 1,252 | .131 | .009 | ns |
| User-Group*Age-Group | 1.094 | 1,252 | .297 | .004 | ns |

*Stop signal Go Trial % Correct: Mann Whitney-U results*

For go trial % correct, it was found that the assumption of normality of residuals was violated. Therefore, we conducted a set of non-parametric Mann Whitney-U tests to further examine these results. This confirmed that controls correctly responded to go trials at a higher proportion than users (*U* = 7202, *p* = .001). In contrast to the ANOVA result, another comparison revealed an effect of age-group, with adults correctly responding to go trials at a higher proportion than adolescents (*U* = 7786.5, *p* = .014). Separate comparisons of adolescent controls vs adolescent users (U = 188877.5, p = .033), and adult controls vs adult controls (*U* = 1717.5, *p* = .007) revealed that in both age-groups, controls correctly responded to a higher proportion of go trials.

|  | **F** | **df** | **p** | **η_p_^2^** | **Group Differences** |
| --- | --- | --- | --- | --- | --- |
| **Prose recall** |  |  |  |  |  |
| User-Group | 4.981 | 1,251 | 0.027 | 0.019 | Controls>users* |
| Age-Group | 7.053 | 1,251 | 0.008 | 0.027 | Adults>adolescents* |
| Time | 139.669 | 1,251 | <0.001 | 0.358 | Immediate>delayed*** |
| User-Group*Age-Group | 2.810 | 1,251 | 0.095 | 0.011 | ns |
| User-Group*Time | 0.291 | 1,251 | 0.590 | 0.001 | ns |
| Age-Group*Time | 2.078 | 1,251 | 0.151 | 0.008 | ns |
| User-Group*Age-Group*Time | 0.054 | 1,251 | 0.816 | <0.001 | ns |

**Table S11**. Full results from 2x2x2 mixed ANOVA of immediate and delayed prose recall, *p<0.05, **p<0.01, ***p<0.001.

.

**Table S12**. Correlations between our primary outcome variables (3-back d prime, stop signal reaction time, delayed recall) and age-of-onset, only in adult users. There were no significant associations.

|  | **r** | **p** | **n** |
| --- | --- | --- | --- |
| **d’ 3-back (N-Back)** | -0.040 | 0.767 | 58 |
| **SSRT (Stop Signal)** | 0.006 | 0.960 | 67 |
| **Delayed Recall (Prose Recall)** | -0.064 | 0.607 | 68 |

**Figure S3**: Scatter plot of cannabis days/week vs delayed prose recall score in adolescent and adult users. Scatter plot shows a trend significant relationship between cannabis days/week and prose recall (delayed) (*F*(1,141)=3.276, *p*=.072, η_p_^2^=0.023), but no interaction between age-group and cannabis days/week.

**Figure S4**: Scatter plot of cannabis days/week vs 3-back d’ in adolescent and adult users. Scatter plot shows no significant relationship between cannabis days/week and d’, and no interaction between age-group and cannabis days/week.

**Figure S5:** Scatter plot of cannabis days/week vs SSRT in adolescent and adult users. Scatter plot shows no significant relationship between cannabis days/week and SSRT, and no interaction between age-group and cannabis days/week.

# **Section 6: Substance Use Variables**

**Table S13**. Lifetime drug use variables for adolescent controls, adolescent users, adult controls and adult users. Use of e-cigarettes in past 12 months is missing for one adolescent user

|  | Adolescent user | Adolescent control | Adult user | Adult control |
| --- | --- | --- | --- | --- |
| Alcohol: ever used | 75 (98.7%) | 61 (96.8%) | 71 (100.0%) | 64 (100.0%) |
| Alcohol: used in past 12 weeks | 71 (93.4%) | 55 (87.3%) | 67 (94.4%) | 61 (95.3%) |
| Tobacco: ever used | 74 (97.4%) | 49 (77.8%) | 66 (93.0%) | 58 (90.6%) |
| Tobacco: used in past 12 weeks | 59 (77.6%) | 18 (28.6%) | 39 (54.9%) | 18 (28.1%) |
| Ecstasy: ever used | 54 (71.1%) | 5 (7.9%) | 52 (73.2%) | 21 (32.8%) |
| Ecstasy: used in past 12 weeks | 35 (46.1%) | 1 (1.6%) | 22 (31.0%) | 2 (3.1%) |
| Laughing gas: ever used | 66 (86.8%) | 22 (34.9%) | 43 (60.6%) | 24 (37.5%) |
| Laughing gas: used in past 12 weeks | 37 (48.7%) | 4 (6.3%) | 5 (7.0%) | 1 (1.6%) |
| E-cigarette: ever used | 50 (65.8%) | 33 (52.4%) | 36 (50.7%) | 16 (25.0%) |
| E-cigarette: used in past 12 weeks | 9 (12.0%) | 10 (15.9%) | 9 (12.7%) | 3 (4.7%) |
| Ketamine: ever used | 48 (63.2%) | 7 (11.1%) | 35 (49.3%) | 10 (15.6%) |
| Ketamine: used in past 12 weeks | 36 (47.4%) | 4 (6.3%) | 10 (14.1%) | 1 (1.6%) |
| LSD: ever used | 29 (38.2%) | 1 (1.6%) | 27 (38.0%) | 3 (4.7%) |
| LSD: used in past 12 weeks | 10 (13.2%) | 0 (0.0%) | 5 (7.0%) | 0 (0.0%) |
| Cocaine: ever used | 28 (36.8%) | 1 (1.6%) | 52 (73.2%) | 18 (28.1%) |
| Cocaine: used in past 12 weeks | 13 (17.1%) | 1 (1.6%) | 23 (32.4%) | 6 (9.4%) |
| Magic mushrooms: ever used | 16 (21.1%) | 1 (1.6%) | 39 (54.9%) | 12 (18.8%) |
| Magic mushrooms: used in past 12 weeks | 3 (3.9%) | 0 (0.0%) | 6 (8.5%) | 3 (4.7%) |
| Alprazolam ‘Xanax’: ever used | 23 (42.6%) | 0 (0.0%) | 10 (17.2%) | 3 (5.0%) |
| Alprazolam ‘Xanax’: used in past 12 weeks | 4 (7.5%) | 0 (0.0%) | 2 (3.4%) | 0 (0.0%) |
| Diazepam ‘Valium’: ever used | 13 (24.1%) | 0 (0.0%) | 12 (21.1%) | 6 (10.0%) |
| Diazepam ‘Valium’: used in past 12 weeks | 3 (5.7%) | 0 (0.0%) | 0 (0.0%) | 4 (1.8%) |
| Amphetamine: ever used | 13 (17.1%) | 1 (1.6%) | 21 (29.6%) | 2 (3.1%) |
| Amphetamine: used in past 12 weeks | 5 (6.6%) | 0 (0.0%) | 4 (5.6%) | 0 (0.0%) |
| Synthetic cannabinoids: ever used | 5 (6.6%) | 0 (0.0%) | 11 (15.5%) | 2 (3.1%) |
| Synthetic cannabinoids: used in past 12 weeks | 0 (0.0%) | 0 (0.0%) | 0 (0.0%) | 0 (0.0%) |
| Methamphetamine: ever used | 0 (0.0%) | 0 (0.0%) | 2 (2.8%) | 1 (1.6%) |
| Methamphetamine: used in past 12 weeks | 0 (0.0%) | 0 (0.0%) | 1 (1.4%) | 0 (0.0%) |
| Heroin: ever used | 0 (0.0%) | 0 (0.0%) | 0 (0.0%) | 1 (1.6%) |
| Heroin: used in past 12 weeks | 0 (0.0%) | 0 (0.0%) | 0 (0.0%) | 0 (0.0%) |
| Crack cocaine: ever used | 0 (0.0%) | 0 (0.0%) | 2 (2.8%) | 0 (0.0%) |
| Crack cocaine: used in past 12 weeks | 0 (0.0%) | 0 (0.0%) | 0 (0.0%) | 0 (0.0%) |

**Table S14**. Cannabis type most commonly used in the last 12 weeks by adolescent users and adult users from the timeline follow-back. Strong herbal cannabis refers to ‘skunk’, typically indoor-grown seedless cannabis, sinsemilla, or ‘high-grade’. Weak herbal cannabis refers to typically outdoor-grown cannabis, which is darker in colour and has seeds in it, and can be called ‘Thaiweed’, ‘brickweed’, ‘ditchweed’. Hash/resin refers to the brown solid which is made by compressing the resin glands/trichomes of the cannabis plant. The one ‘other’ was ‘THC oil’. The groups did not differ in their most common cannabis type (χ_3_^2^=3.866, p=0.276).

|  | Adolescent User | Adult User |
| --- | --- | --- |
| Strong herbal (i.e. ‘skunk’) | 69 (90.8%) | 59 (83.1%) |
| Weak herbal | 0 (0.0%) | 2 (2.8%) |
| Hash/resin | 7 (9.2%) | 9 (12.7%) |
| Other | 0 (0.0%) | 1 (1.4%) |
|  |  |  |

# **Section 7: Supplementary Discussion**

*Verbal episodic memory*

One previous cross-sectional study comparing adolescent controls, alcohol users and cannabis users reported a delayed recall user impairment with a large effect size (d=0.84), which remained significant after adjusting for covariates (Solowij et al., 2011). However, they did not include SES or premorbid IQ as covariates, although their groups were matched on these variables (Solowij et al., 2011).

We did not find strong evidence for a differential relationship between cannabis use and VEM in adolescents and adults, while previous work found that people with an earlier age of cannabis use onset recalled less words than those with a later age of onset (Becker et al., 2018; Solowij et al., 2011). This may be related to the different study design. In our study, adolescent users had earlier ages of onset and shorter durations of use than adult users; while in previous research, adult participants with an earlier age of onset had longer durations of use. The characteristics of these previous studies, and others like them, may bias findings in favour of negative associations between age-of-onset and impaired outcomes.

*Response inhibition*

Our primary outcome variable, SSRT, showed no relationship with user-group or age-group, or their interaction. However, when considering the secondary outcome variables, the groups displayed different patterns of behaviour. The users, compared to the controls, were less accurate on go trials but more accurate on stop trials. As we pre-specified our primary outcome variable as the SSRT, based on the task design (Verbruggen & Logan, 2008), and the secondary outcome differences were small, we do not interpret these differences meaningful.

*Association with cannabis use frequency*

In our analysis of the user-group, we found only a trend association between cannabis use frequency and delayed recall. In contrast, Solowij and colleagues (Solowij et al., 2011) found a strong negative correlation cannabis use frequency and delayed recall, where a greater frequency of cannabis use was significantly associated with poorer recall. Similarly, a meta-analysis found that heavy users, who used cannabis over 20 times a month, performed significantly worse on a collection of memory tasks (including VEM) than moderate or light users (Schoeler et al., 2016). Collectively, these results suggest more frequent cannabis use is associated with worse VEM.

*Change in cannabis strength over the last ten years*

On average, adult users initiated cannabis use nine years before they started the study, in 2008-2010. In contrast, adolescent users initiated cannabis use two and a half years before they started the study, in 2015-2017. During that time, the strength of cannabis (i.e. its THC concentration, or potency) has increased (Freeman et al., 2019, 2021) In the UK, THC concentration in strong herbal cannabis (i.e. high-strength herbal cannabis, ‘skunk’, sinsemilla) increased from approximately 11% in 2008 to approximately 15% in 2016, and THC concentration in hash increased from approximately 5% in 2008 to 13% in 2016 (Freeman et al., 2019). Furthermore, the dominance of strong herbal cannabis, relative to low-strength herbal cannabis and hash, increased between 2005 and 2016 (Potter et al., 2008, 2018). Thus, the adult users likely used lower strength cannabis when they initiated use than when adolescent users initiated use. These differences in the history of cannabis use should be kept in mind when contrasting the adolescent and adult users. However, when the participants took part in the study, a similar number of adolescent and adult users used strong herbal cannabis (i.e. ‘skunk’) (see table 2 and S14). Furthermore, albeit in a small sample, the average cannabis strength, in terms of THC concentration, was remarkably similar in both groups (mean=21%).

*Further limitations given group differences*

Despite this recent increase in cannabis strength, and the greater self-reported quantity of cannabis used on a day of use by adolescents, given the longer average duration of weekly cannabis use in the adult user group compared to the adolescent user group, it is likely the total amount of lifetime THC consumed was greater in the adult user group than the adolescent user group.

These group differences are potential limitations of our cross-sectional study. It is theoretically possible that the putatively detrimental effect of early cannabis use in adolescent users was cancelled out by the putatively detrimental effect of a longer duration of regular cannabis use in adult users. In opposition to this claim, we did not find any significant associations between age of cannabis onset and cognitive performance in the adult user group. Furthermore, our user groups did not differ from their age-matched controls on response inhibition or SWM, so the concern that adolescent vs. adult cannabis use might be lost through different cannabis-related histories is less relevant. Subsequent longitudinal analyses will help to throw light on the impact of current adolescent use in a group of people who started using at 14.5 years vs. the impact of current adult cannabis use in a group of people who started using at 18 years.

**References**

Adamson, S. J., Kay-Lambkin, F. J., Baker, A. L., Lewin, T. J., Thornton, L., Kelly, B. J., & Sellman, J. D. (2010). An improved brief measure of cannabis misuse: the Cannabis Use Disorders Identification Test-Revised (CUDIT-R). *Drug and Alcohol Dependence*, *110*(1–2), 137–143.

Becker, M. P., Collins, P. F., Schultz, A., Urošević, S., Schmaling, B., & Luciana, M. (2018). Longitudinal changes in cognition in young adult cannabis users. *Journal of Clinical and Experimental Neuropsychology*, *40*(6), 529–543.

Bohn, M. J., & Babor, H. R. K. (1995). The Alcohol Use Disorders Identification Test (AUDIT): validation of a screening instrument for use in medical settings. *Journal of Studies on Alcohol and Drugs*, *56*(4), 423–432.

Booth, J. R., Burman, D. D., Meyer, J. R., Lei, Z., Trommer, B. L., Davenport, N. D., Li, W., Parrish, T. B., Gitelman, D. R., & Mesulam, M. M. (2003). Neural development of selective attention and response inhibition. *NeuroImage*, *20*(2), 737–751. https://doi.org/10.1016/S1053-8119(03)00404-X

Broyd, S. J., van Hell, H. H., Beale, C., Yuecel, M., & Solowij, N. (2016). Acute and chronic effects of cannabinoids on human cognition—a systematic review. *Biological Psychiatry*, *79*(7), 557–567.

Cowan, N., Saults, J. S., & Morey, C. C. (2006). Development of working memory for verbal–spatial associations. *Journal of Memory and Language*, *55*(2), 274–289.

Curry, J. F., Logue, P. E., & Butler, B. (1986). Child and adolescent norms for Russell’s revision of the Wechsler Memory Scale. *Journal of Clinical Child Psychology*, *15*(3), 214–220.

De Haan, L., Kuipers, E., Kuerten, Y., Van Laar, M., Olivier, B., & Cornelis Verster, J. (2011). The rT-18: a new screening tool to assess young adult risk-taking behavior. *International Journal of General Medicine*.

Duperrouzel, J. C., Hawes, S. W., Lopez-Quintero, C., Pacheco-Colón, I., Coxe, S., Hayes, T., & Gonzalez, R. (2019). Adolescent cannabis use and its associations with decision-making and episodic memory: Preliminary results from a longitudinal study. *Neuropsychology*, *33*(5), 701.

Freeman, T. P., Craft, S., Wilson, J., Stylianou, S., ElSohly, M., di Forti, M., & Lynskey, M. T. (2021). Changes in delta‐9‐tetrahydrocannabinol (THC) and cannabidiol (CBD) concentrations in cannabis over time: systematic review and meta‐analysis. *Addiction*, *116*(5), 1000–1010.

Freeman, T. P., Groshkova, T., Cunningham, A., Sedefov, R., Griffiths, P., & Lynskey, M. T. (2019). Increasing potency and price of cannabis in Europe, 2006–16. *Addiction*, *114*(6), 1015–1023.

Freeman, T. P., & Winstock, A. R. (2015). Examining the profile of high-potency cannabis and its association with severity of cannabis dependence. *Psychological Medicine*, *45*(15), 3181–3189.

Haatveit, B. C., Sundet, K., Hugdahl, K., Ueland, T., Melle, I., & Andreassen, O. A. (2010). The validity of d prime as a working memory index: Results from the Bergen n-back task. *Journal of Clinical and Experimental Neuropsychology*, *32*(8), 871–880. https://doi.org/10.1080/13803391003596421

Harvey, M. A., Sellman, J. D., Porter, R. J., & Frampton, C. M. (2007). The relationship between non-acute adolescent cannabis use and cognition. *Drug and Alcohol Review*, *26*(3), 309–319. https://doi.org/10.1080/09595230701247772

Holdnack, H. A. (2001). Wechsler test of adult reading: WTAR. *San Antonio, TX: The Psychological Corporation*.

Jacobus, J., Squeglia, L. M., Infante, M. A., Castro, N., Brumback, T., Meruelo, A. D., & Tapert, S. F. (2015). Neuropsychological performance in adolescent marijuana users with co-occurring alcohol use: A three-year longitudinal study. *Neuropsychology*, *29*(6), 829.

Lawn, W., Mokrysz, C., Borissova, A., Lees, R., Petrilli, K., Bloomfield, M., Wall, M., Freeman, T., & Curran, V. (2020). *STUDY PROTOCOL CANNTEEN: HOW DOES LONG-TERM CANNABIS USE AFFECT TEENAGERS’ AND ADULTS’ COGNITION, MENTAL HEALTH AND BRAINS*. OSF.

Luna, B., Padmanabhan, A., & O’Hearn, K. (2009). What has fMRI told us about the Development of Cognitive Control through Adolescence? *Brain and Cognition*, *72*, 101–113.

Matzke, D., Verbruggen, F., & Logan, G. D. (2018). The Stop-Signal Paradigm. In *Stevens’ Handbook of Experimental Psychology and Cognitive Neuroscience*. https://doi.org/10.1002/9781119170174.epcn510

Potter, D. J., Clark, P., & Brown, M. B. (2008). Potency of Δ9–THC and other cannabinoids in cannabis in England in 2005: Implications for psychoactivity and pharmacology. *Journal of Forensic Sciences*, *53*(1), 90–94.

Potter, D. J., Hammond, K., Tuffnell, S., Walker, C., & di Forti, M. (2018). Potency of Δ9–tetrahydrocannabinol and other cannabinoids in cannabis in England in 2016: Implications for public health and pharmacology. *Drug Testing and Analysis*, *10*(4), 628–635.

Robinson, S. M., Sobell, L. C., Sobell, M. B., & Leo, G. I. (2014). Reliability of the Timeline Followback for cocaine, cannabis, and cigarette use. *Psychology of Addictive Behaviors*. https://doi.org/10.1037/a0030992

Schoeler, T., Kambeitz, J., Behlke, I., Murray, R., & Bhattacharyya, S. (2016). The effects of cannabis on memory function in users with and without a psychotic disorder: findings from a combined meta-analysis. *Psychological Medicine*, *1*, 177–188.

Schutte, A. R., & Spencer, J. P. (2009). Tests of the Dynamic Field Theory and the Spatial Precision Hypothesis: Capturing a Qualitative Developmental Transition in Spatial Working Memory. *Journal of Experimental Psychology: Human Perception and Performance*, *35*(6), 1698–1725. https://doi.org/10.1037/a0015794

Schutte, A. R., Spencer, J. P., & Schöner, G. (2003). Testing the Dynamic Field Theory: Working Memory for Locations Becomes More Spatially Precise over Development. *Child Development*, *74*(5), 1393–1417. https://doi.org/10.1111/1467-8624.00614

Solowij, N., Jones, K. A., Rozman, M. E., Davis, S. M., Ciarrochi, J., Heaven, P. C. L., Lubman, D. I., & Yücel, M. (2011). Verbal learning and memory in adolescent cannabis users, alcohol users and non-users. *Psychopharmacology*, *216*(1), 131–144.

Sowell, E. R., Delis, D., Stiles, J., & Jernigan, T. L. (2001). Improved memory functioning and frontal lobe maturation between childhood and adolescence: a structural MRI study. *Journal of the International Neuropsychological Society*, *7*(3), 312–322.

Tamm, L., Menon, V., & Reiss, A. L. (2002). Maturation of brain function associated with response inhibition BrowZine Journal Cover. *Journal of the American Academy of Child & Adolescent Psychiatry*, *41*(10), 1231–1238. https://doi.org/10.1097/01.CHI.0000020272.43550.5E

Tervo-Clemmens, B., Simmonds, D., Calabro, F. J., Day, N. L., Richardson, G. A., & Luna, B. (2018). Adolescent cannabis use and brain systems supporting adult working memory encoding, maintenance, and retrieval. *NeuroImage*, *169*(January 2017), 496–509. https://doi.org/10.1016/j.neuroimage.2017.12.041

Vakil, E., Greenstein, Y., & Blachstein, H. (2010). Normative data for composite scores for children and adults derived from the Rey Auditory Verbal Learning Test. *The Clinical Neuropsychologist*, *24*(4), 662–677.

Verbruggen, F., Aron, A. R., Band, G. P. H., Beste, C., Bissett, P. G., Brockett, A. T., Brown, J. W., Chamberlain, S. R., Chambers, C. D., Colonius, H., Colzato, L. S., Corneil, B. D., Coxon, J. P., Dupuis, A., Eagle, D. M., Garavan, H., Greenhouse, I., Heathcote, A., Huster, R. J., … Boehler, C. N. (2019a). A consensus guide to capturing the ability to inhibit actions and impulsive behaviors in the stop-signal task. *ELife*, *8*, 1–26. https://doi.org/10.7554/eLife.46323

Verbruggen, F., Aron, A. R., Band, G. P. H., Beste, C., Bissett, P. G., Brockett, A. T., Brown, J. W., Chamberlain, S. R., Chambers, C. D., Colonius, H., Colzato, L. S., Corneil, B. D., Coxon, J. P., Dupuis, A., Eagle, D. M., Garavan, H., Greenhouse, I., Heathcote, A., Huster, R. J., … Boehler, C. N. (2019b). A consensus guide to capturing the ability to inhibit actions and impulsive behaviors in the stop-signal task. *ELife*, *8*, 1–26. https://doi.org/10.7554/eLife.46323

Verbruggen, F., & Logan, G. D. (2008). Response inhibition in the stop-signal paradigm. *Trends in Cognitive Sciences*, *12*(11), 418–424.
